# Fairy “tails”: flexibility and function of intrinsically disordered extensions in the photosynthetic world

**DOI:** 10.3389/fmolb.2015.00023

**Published:** 2015-05-19

**Authors:** Gabriel Thieulin-Pardo, Luisana Avilan, Mila Kojadinovic, Brigitte Gontero

**Affiliations:** UMR 7281, Centre National de la Recherche Scientifique, Aix-Marseille UniversitéMarseille, France

**Keywords:** intrinsically disordered proteins, GAPDH, CP12, Rubisco activase, cryptochromes, transcription factors

## Abstract

Intrinsically Disordered Proteins (IDPs), or protein fragments also called Intrinsically Disordered Regions (IDRs), display high flexibility as the result of their amino acid composition. They can adopt multiple roles. In globular proteins, IDRs are usually found as loops and linkers between secondary structure elements. However, not all disordered fragments are loops: some proteins bear an intrinsically disordered extension at their C- or N-terminus, and this flexibility can affect the protein as a whole. In this review, we focus on the disordered N- and C-terminal extensions of globular proteins from photosynthetic organisms. Using the examples of the A_2_B_2_-GAPDH and the α Rubisco activase isoform, we show that intrinsically disordered extensions can help regulate their “host” protein in response to changes in light, thereby participating in photosynthesis regulation. As IDPs are famous for their large number of protein partners, we used the examples of the NAC, bZIP, TCP, and GRAS transcription factor families to illustrate the fact that intrinsically disordered extremities can allow a protein to have an increased number of partners, which directly affects its regulation. Finally, for proteins from the cryptochrome light receptor family, we describe how a new role for the photolyase proteins may emerge by the addition of an intrinsically disordered extension, while still allowing the protein to absorb blue light. This review has highlighted the diverse repercussions of the disordered extension on the regulation and function of their host protein and outlined possible future research avenues.

## Introduction

Proteins occupy a central position in the architecture and functioning of living matter. A major objective of protein biochemistry consists in explaining the physiological functions of these molecules by means of structural studies, also known as the “structure-function” relationship. Among others, X-ray crystallography is a powerful tool to solve macromolecular three-dimensional 3D structures. However, some proteins cannot be crystallized because they are fully disordered or possess disordered parts that are missing in the electron density map of the crystals. In the 1990s, Sedzik and Kirschner (1992) attempted to crystallize the myelin basic protein (MBP), the predominant extrinsic protein in both central and nervous system myelins. After several attempts, the authors concluded that MBP adopts a random coil conformation and that as long as its flexibility was not suppressed, it was not possible to obtain crystals (Sedzik and Kirschner, 1992). MBP was one of the first examples of many other “un-crystallizable” proteins. These proteins, originally named Intrinsically Unstructured Proteins (IUPs), are nowadays termed Intrinsically Disordered Proteins (IDPs) (Wright and Dyson, 1999; Dunker et al., 2001, 2008a,b). In 1998, Romero et al. showed that 15 000 proteins from the Swiss-Prot database contain one or more Intrinsically disordered regions (IDRs) comprising more than 40 amino acid residues (Romero et al., 1998). It was shown later that despite their lack of well-defined 3D structure, many partially or completely disordered proteins are functional (Wright and Dyson, 1999; Dunker et al., 2001, 2008a,b; Tompa, 2002). In the late 1990s, studies of disordered yet functional proteins emerged as a new research field, extending the traditional paradigm to include a more comprehensive view of protein structure-function (Wright and Dyson, 1999; Dunker et al., 2001; Tompa, 2002; Dunker et al., 2008a,b). In the past, different models have been proposed to explain protein functioning, and protein flexibility has appeared as a key point (Fersht, 1998). Among these models, the “induced-fit” model (Koshland et al., 1966) introduced the idea that protein conformational changes could be triggered upon ligand binding. These notions were applied to IDPs, and many of them were shown to undergo an “induced-folding” upon binding to their partners (Dunker et al., 2002). Short motifs called MoREs (Molecular Recognition Elements) are often involved in the interaction, involving disorder to order transitions (Fuxreiter et al., 2004, 2007; Oldfield et al., 2005; Mohan et al., 2006; Vacic et al., 2007; Hazy and Tompa, 2009). However, the idea that preformed binding elements exist before the binding, and even in the absence of a partner, led to the “conformational selection” model. In some cases, the IDP is not fully structured in the presence of its partner and the term “fuzziness” was coined by Fuxreiter and Tompa to describe such complexes (Tompa and Fuxreiter, 2008; Hazy and Tompa, 2009; Fuxreiter and Tompa, 2012). The flexibility of IDPs increases the chance of their polypeptide chains adopting the right conformations in the presence of their partners. Furthermore, the high ratio of hydrophilic residues in IDPs facilitates initial contacts with their partners. The interactions are also stronger with IDPs: their lack of structure or absence of rigidity increasing association constants (Dunker et al., 2001; Meszaros et al., 2007; Chouard, 2011). The ability of the IDPs to adopt multiple conformations allows the same region to adapt to different binding sites in many “induced-fits” and thus to have multiple partners (Uversky et al., 2000; Tompa, 2002; Uversky, 2002, 2011; Meszaros et al., 2007; Carmo-Silva and Salvucci, 2013). The discovery of IDPs and their singular lack of definite structure brought nuances to the “structure-function” dogma, showing that the same structure, or lack of one, could have multiple partners and thus multiple functions (Wright and Dyson, 1999; Dunker et al., 2001; Tompa, 2002; Uversky, 2002, 2011; Meszaros et al., 2007; Sun et al., 2013). In this regard, IDPs could be seen as the “master keys” of the protein-protein interaction network.

The ability of IDPs to bind to multiple partners makes them naturally good regulators, as they can modulate the activity of several proteins in a coordinated way (Dunker et al., 2001, 2002, 2005; Gavin et al., 2002, 2006; Haynes et al., 2006; Patil and Nakamura, 2006; Mittag et al., 2010; Uversky, 2010; Pancsa and Tompa, 2012). Therefore, multiple areas of the cellular response can be affected by a single signal allowing IDPs to play a major role in regulatory pathways (Tompa, 2002; Dunker et al., 2005; Haynes et al., 2006; Uversky, 2010; Pancsa and Tompa, 2012). The flexibility of IDPs can also be modified by the cellular environment or by post-translational modifications. IDPs and IDRs are often targets of different post-translational modifications (the most common being phosphorylation, methylation and ubiquitination) which can radically affect their affinity for their partners and their stability, thus multiplying the possibilities for a fine-tuned regulation (Tompa, 2002; Dunker et al., 2005; Haynes et al., 2006; Uversky, 2010; Pancsa and Tompa, 2012). These particularities make IDPs the hubs in a vast net of protein-protein interactions (Gavin et al., 2002, 2006). They carry out basic functions such as regulation of metabolic pathways, transcription, translation or cellular signal transduction; they can act as scavengers of toxic molecules and they play a key role in the assembly of multi-protein complexes (Uversky, 2011). Moreover, their roles in several diseases of major medical interest, such as cancer (Castillo et al., 2014; Saha et al., 2014; Xue et al., 2014), Alzheimer's disease (Uversky, 2009; Kovacech and Novak, 2010; Salminen et al., 2011; Karagoz and Rudiger, 2015) prion disease (Tompa, 2009; Uversky, 2009; Breydo and Uversky, 2011) or Parkinson's disease (Uversky, 2009; Hazy et al., 2011; Breydo et al., 2012; Alderson and Markley, 2013) have been extensively studied (Babu et al., 2011).

While the discovery and characterization of IDPs and IDRs is a rapidly growing, and an increasingly recognized, area of protein science, (Tompa, 2002; Uversky, 2010; Uversky and Dunker, 2010; Chouard, 2011), little information is available photosynthetic organisms, where IDPs have been described as central players in many responses such as biotic and abiotic stress, development, metabolism regulation, or adaptation to oxic atmosphere (Kragelund et al., 2012; Pancsa and Tompa, 2012; Yruela and Contreras-Moreira, 2012, 2013; Pietrosemoli et al., 2013; Sun et al., 2013; Panda and Ghosh, 2014). Published data mainly concern *Arabidopsis thaliana*, a higher plant model with one of the best-annotated sequenced genomes (Arabidopsis Genome Initiative, 2000). Yet, the recent analysis of 12 plant genomes revealed that the occurrence of disorder in plants is similar to the one found in other eukaryotes (Bracken et al., 1999; Yruela and Contreras-Moreira, 2012, 2013; Sun et al., 2013). An *in silico* analysis of plant nuclear proteomes suggested a higher disorder in the internal part of nuclear-encoded plant proteins rather than at their extremities, in contrast to the chloroplast- and mitochondrion-encoded proteomes (Yruela and Contreras-Moreira, 2012). This is also pointed by studies on prokaryotes showing that the IDRs may be more frequent at the extremities of the proteins that act as “molecular shields” such as chaperones (Krisko et al., 2010; Chakrabortee et al., 2012).

In this review, we describe several globular proteins with N- or C-terminal IDR extensions in photsynthetic organisms, as opposed to entirely disordered proteins or globular proteins containing one or more IDRs in the middle of their sequences. The aim of this work is not to give an exhaustive list of the roles undertaken by such disordered extensions, as this has recently been reviewed (Uversky, 2013). Instead, we focus on globular proteins or domains that acquired their disordered tails during evolution, using examples from photosynthetic organisms. The addition of a disordered extension to a globular protein created new regulation opportunities, making these proteins responsive to environmental factors through self-regulation, post-translational modifications or new protein-protein interactions. We illustrate the impact of disordered extensions by describing proteins involved in photosynthetic metabolism and regulation of gene expression (Table 1).

**Table 1:** **Summary of the characteristics of the Intrinsically disordered extensions presented in this review**.

	**Protein/Protein family**	**Protein role**	**Position of the extension**	**Role (or possible role) of the disordered extension**	Particularities of the disordered extension^*^	**References**
Metabolism regulation	GAPDH GapB	Enzyme from the Benson-Calvin cycle	C-terminus	Autonomous redox regulation of the GAPDH activity	Pair of redox-sensitive cysteine residues	Cerff, 1979; Brinkmann et al., 1989; Baalmann et al., 1996; Li and Anderson, 1997; Scagliarini et al., 1998; Sparla et al., 2002; Petersen et al., 2006
	Rubisco Actlvase (α isoform)	Activator of the Rubisco enzyme	C-terminus	Possible redox regulation of the RCA activity	Pair of redox-sensitive cysteine residues	Werneke et al., 1989; Shen and Ogren, 1992; Zhang and Portis, 1999; Portis, 2003; Henderson et al., 2011; Stotz et al., 2011; Carmo-Silva and Salvucci, 2013; Gontero and Salvucci, 2014
Gene regulation	NAC family (No Apical Meristem, ATAF, Cup Shaped Cotyledon)	Transcription factors	C-terminus	Regulation of the NAC transcription factor domain through protein-protein interactions	Presence of multiple MoREs (conserved within a subfamily)	Ooka et al., 2003; Olsen et al., 2005; Jensen et al., 2010a,b; Kjaersgaard et al., 2011
	bZIP family (basic Leucine Zipper)	Transcription factors	N-terminus	Regulation of the bZIP transcription factor domain and its stability through protein-protein interactions and post-translational modifications	Presence of multiple MoREs (conserved within a subfamily) Phosphorylation sites	Ang et al., 1998; Campbell et al., 2000; Hardtke et al., 2000; Moreau et al., 2004; Yoon et al., 2006; Sun et al., 2013
	TCP family [Teosinte branched 1 (tbl), Cycloidea (cyc) and Proliferating Cell Factor	Transcription factors	N-terminus and C-terminus	N-terminus: Binding of target DNA C-tenninus: TCP self-association and regulation	N-terminus: Induced-fit binding of DNA C-terminus: Coiled-coil self association	Viola et al., 2011, 2012; Steiner et al., 2012; Valsecchi et al., 2013
Signaling	GRAS family [Gibberellic Acid Insensitive (GAI). Repressor of Gai (RGA) and Scarecrow (SCR)]	Transcriptional co-activator	N-terminus	Regulation of the GRAS activator domain and its stability through protein-protein interactions and post-translational modifications	Presence of multiple MoREs (conserved within a subfamily) Phosphorylation sites	Triezenberg, 1995; Czikkel and Maxwell, 2007; Sun et al., 2010, 2011, 2012, 2013
	Cryptochiomes	Light-signaling Control of circadian and annual cyles	C-terminus	Protein-protein interaction upon captation of blue light: Initiation of developpemental responses	Presence of MoREs Multiple phosphorylation sites	Lin and Shalitin, 2003; Green, 2004; Partch et al., 2005; Yu et al., 2010; Chaves et al., 2011; Liu et al., 2011a,b

* *All the extensions present features of “disorder”: enrichment in hydrophylic charged amino acids and few hydrophobic residues*.

## GAPDH and CP12

As knowledge about proteins progressed, new results appeared showing that some enzymes were able to carry out more than one function in a single polypeptide chain and were classified as multifunctional proteins (Kirschner and Bisswanger, 1976). In many cases, the dual function resulted from the fusion of two genes that initially encoded different proteins. Later on, the term “moonlighting” (Jeffery, 1999) categorized proteins that have different functions. The glyceraldehyde-3-phosphate dehydrogenase (GAPDH) is a well-known moonlighting enzyme and has at least ten distinct, confirmed non-enzymatic activities apart from its enzymatic function (Sirover, 1999, 2011; Hildebrandt et al., 2015). The GAPDHs constitute a large and diverse family of dehydrogenases universally represented in living organisms. They catalyze the reductive dephosphorylation of 1, 3-bisphosphoglyceric acid (BPGA) producing glyceraldehyde-3-phosphate (GAP) and inorganic phosphate using NAD(P)H as a cofactor (Trost et al., 2006). Glycolytic GAPDHs(also named GapC) are NAD-specific and mainly found in the cytosol, but in land plants a second type of glycolytic GAPDH named GapCp is targeted to the chloroplast(Petersen et al., 2003; Marri et al., 2005; Munoz-Bertomeu et al., 2010). Both GapC and GapCp are NAD-specific and form homotetramers *in vivo* that are not subject to complex regulatory mechanisms. However, in photosynthetic organisms, another GAPDH catalyzes the unique reductive step of the Benson-Calvin cycle is present and uses both NADH and NADPH with a marked preference for NADPH (Falini et al., 2003).

**Figure 1:**
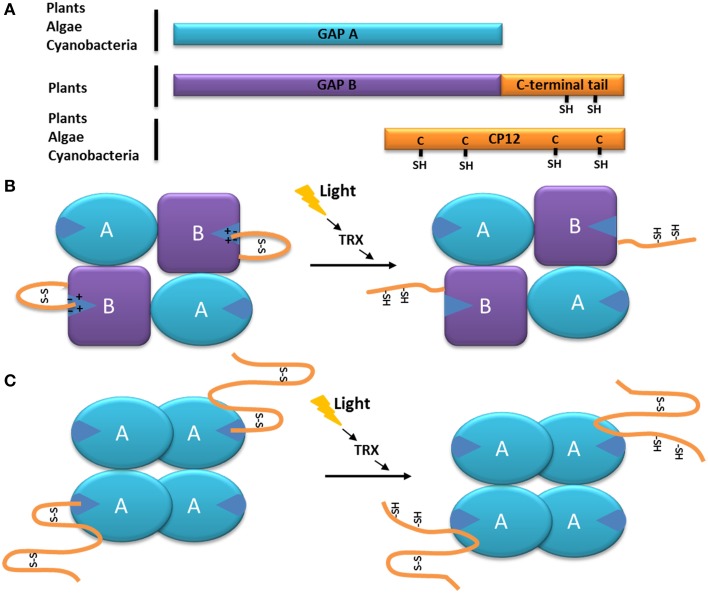
**Model of the function of the C-terminal extension of the GAPDH subunit GapB. (A)** Schematic organization of the GapB from higher plants and *O. tauri* (Robbens et al., 2007) subunit compared to the GapA and CP12 proteins. The C-terminal extension of GapB is homologous to the C-terminal of CP12 and present two regulatory cysteine residues. **(B)** Schematic representation of the autonomous redox regulation of the A_2_B_2_-GAPDH. When oxidized, the C-terminal extension of the GapB subunit presents a disulfide bridge, which places the C-terminal amino acids inside the active site of the enzyme, resulting in its inhibition. The disulfide bridge can be reduced by the thioredoxin *f* (TRX), and the enzyme becomes active. **(C)** Schematic representation of the autonomous redox regulation of the A_4_-GAPDH by CP12. When oxidized, the C-terminal part of the CP12 protein presents a disulfide bridge, which places its C-terminal amino acids inside the active site of GAPDH, resulting in its inhibition. The disulfide bridge can be reduced by the thoioredoxin *f* (TRX) or DTT and the enzyme becomes active.

Like all GAPDHs, the NADPH-dependent GAPDH is made up of two functional domains, one corresponding to the catalytic domain (residues 148–313 in spinach GAPDH) and the other one being the cofactor-binding domain, or Rossman fold (residues 1–147 and 313–334, respectively). The latter contains a flexible, arginine-rich region called the S-loop (Fermani et al., 2001). In higher plants, this GAPDH exists in different forms such as a heterotetramer made up of two GapA and two GapB subunits (A_2_B_2_), a homotetramer made up of four GapA subunits, and as a hexadecamer (A_8_B_8_) (Baalmann et al., 1994, 1995; Scheibe et al., 2002; Howard et al., 2011a,b). The GapB subunit is similar to the GapA subunit but bears a C-terminal extension which has a regulatory function (Cerff, 1979; Brinkmann et al., 1989; Baalmann et al., 1996; Li and Anderson, 1997; Scagliarini et al., 1998; Sparla et al., 2002) (Figure 1A). This subunit is thought to derive from a gene duplication event which occurred near the origin of Streptophyta (which include charophytes and land plants) (Petersen et al., 2006) and might be a construct of a GapA moiety fused at the C-terminus with the C-terminal half of the CP12, a 8.2–8.5 kDa Chloroplast Protein (Pohlmeyer et al., 1996). The portion of CP12 acquired by GapB subunits confers redox properties to GapB-containing GAPDH (Pupillo and Piccari, 1973; Pupillo and Giuliani Piccari, 1975; Wolosiuk and Buchanan, 1976, 1978; Trost and Pupillo, 1993; Baalmann et al., 1994, 1995; Sparla et al., 2002). Cyanobacteria, primitive photosynthetic eukaryotes (including the glaucophyte *Cyanophora paradoxa*), and red and green algae (except charophytes and sister lineages) seem to contain exclusively the GapA subunit (Petersen et al., 2006; Trost et al., 2006). However, the small prasinophyte green alga *Ostreococcus tauri* has been shown to possess GapA and GapB (Robbens et al., 2007).

CP12 is a protein of about 80 amino acid residues that was originally described by Pohlmeyer et al. (1996) and has been found in most photosynthetic organisms (Groben et al., 2010; Gontero and Maberly, 2012; Gontero and Salvucci, 2014; Lopez-Calcagno et al., 2014). The CP12 proteins show high primary sequence variability, in particular at the N-terminus. However, they share some remarkable common features.CP12 proteins have an amino acid composition poor in order-promoting residues although they contain cysteine residues (Groben et al., 2010), and behave abnormally under gel electrophoresis and size exclusion chromatographies (Gontero and Maberly, 2012) suggesting that they are IDPs. Moreover, recent data from fluorescence correlation spectroscopy (FCS) show that the hydrodynamic radius of CP12 from the green alga *Chlamydomonas reinhardtii* is large compared to that expected for globular proteins of this molecular mass (Moparthi et al., 2014). The cysteine residues are involved in the formation of disulfide bridges and peptide loops and are found as pairs at the C-terminus and/or at the N-terminus. When oxidized, CP12 proteins may present α helices maintained by the N-terminal disulfide bridge (Graciet et al., 2003a; Gardebien et al., 2006). The algal CP12 is a key component of a supra-molecular complex controlling the activity of the Benson-Calvin cycle by regrouping several key enzymes of the cycle, including GAPDH, phosphoribulokinase (PRK) and fructose 1,6-bisphosphate aldolase (FBP aldolase). Within the ternary GAPDH/CP12/PRK complex, both enzymes are strongly inhibited (Avilan et al., 1997; Lebreton et al., 1997; Graciet et al., 2003a; Erales et al., 2008; Marri et al., 2008). CP12 forms a fuzzy complex with the green alga *C. reinhardtii* A_4_-GAPDH, as revealed by EPR studies (Mileo et al., 2013) (see the Minireview by Lebreton et al. in this Topic Research). The ternary complex has also been found in the cyanobacterium *Synechococcus elongatus* and in the higher plant *A. thaliana*. The A_4_-GAPDH-CP12 sub-complex from these organisms have been crystallized but in both complexes, the first 50 amino acid residues were not visible in the density map, consistent with a high flexibility of this region in the crystal (Avilan et al., 2000; Matsumura et al., 2011; Fermani et al., 2012). Recently, it was also observed using FCS and FRET (Förster Resonance Energy Transfer) that the algal CP12 flexibility is not abolished by its interactions with either GAPDH or with PRK (Moparthi et al., 2014, 2015).

In the case of the GapB subunit of GAPDH from higher plants, the C-terminal end of the protein (*ca* 30 residues)is strongly homologous to the C-terminal part of CP12 (Pohlmeyer et al., 1996; Trost et al., 2006; Groben et al., 2010) (Figure 1A). The regulation of the *A. thaliana* A_2_B_2_-GAPDH activity by the C-terminal extension of the GapB subunit is now very well understood: upon oxidation (which happens during the day-night transition) the two cysteine residues of the CP12-like tail form a disulfide bridge that places the C-terminal ultimate glutamate residue (E362) inside the active site (stabilized by the electrostatic interactions with an arginine residue R77 involved in the NADP cofactor binding). Consequently, the NADPH cofactor is not able to enter the catalytic site and thus NAPDH-dependant A_2_B_2_-GAPDH activity is inhibited (Sparla et al., 2005; Fermani et al., 2007) (Figure 1B). In contrast, during the night-day transition, the disulfide bridge maintaining the C-terminal extension into the active site is reduced by thioredoxin *f*, thereby releasing the CP12-like tail and resulting in A_2_B_2_-GAPDH activity (Figure 1B) (Sparla et al., 2002; Trost et al., 2006; Fermani et al., 2007). This mechanism is very similar to the one observed in *C. reinhardtii* between the homotetrameric A_4_-GAPDH and free CP12, where the penultimate glutamate (E79) of the CP12 interacts with the arginine residue R82 of A_4_-GAPDH (Figure 1C) (Trost et al., 2006; Erales et al., 2011; Avilan et al., 2012). The reduction of the GAPDH-CP12 by dithiothreitol (DTT) in the alga results in a more active NADPH-GAPDH as a consequence of the rupture of disulfide bridges on CP12. Of interest, DTT, *in vitro* mimicks thioredoxins *in vivo* and it has been shown that CP12 can be reduced by thioredoxin *f* in the light (Marri et al., 2009).

In the higher plant, *A. thaliana* and in the green alga, *C. reinhardtii*, the stoichiometry of the oxidized A_4_-GAPDH-CP12 sub-complex is two CP12 molecules for one A_4_-GAPDH (Marri et al., 2008; Kaaki et al., 2013), while four CP12 molecules interact with each GAPDH tetramer in the cyanobacterium, *S. elongatus* (Matsumura et al., 2011). When interacting with CP12, A_4_-GAPDH activity decreased by two-fold (in the case of the *C. reinhardtii* proteins, the catalytic constant *k_cat_* of the free enzyme was 430 ± 17 s^−1^, and became 251 ± 9 s^−1^ in the presence of CP12), suggesting that only two of the four active sites were blocked (Graciet et al., 2003b) (Figure 1C). The same observation was made for the *A. thaliana* A_2_B_2_-GAPDH: upon oxidation, the A_2_B_2_-GAPDH activity decreased by 2-fold (its *k_cat_* changed from 59 ± 19 s^−1^ to 27 ± 10 s^−1^) although its catalytic constant in a reduced state (*k_cat_* = 59 ± 19 s^−1^) was comparable to the one of the free A_4_-GAPDH (*k_cat_* = 61 ± 4 s^−1^) (Sparla et al., 2004). The regulation of the plant A_2_B_2_-GAPDH and of the algal A_4_-GAPDH-CP12 complex is thus very similar. With the addition of the C-terminal extension within the GapB subunit, the A_2_B_2_-GAPDH has become autonomously redox-regulated, a property that was previously provided through interaction with CP12.

Although the appearance of the GapB subunit represents an important step in the evolution of the redox control of the Calvin-Benson cycle enzymes, this new autonomous regulation co-exists with the CP12-based one in higher plants (Scheibe et al., 2002), and a A_2_B_2_-GAPDH-PRK complex entirely devoid of CP12 has yet to be identified. The presence of CP12 is likely to be required for the assembly of larger supramolecular complex, and in *C. reinhardtii*, A_4_-GAPDH-CP12-PRK was shown to interact with the aldolase (Erales et al., 2008). In this regard, one may wonder how this system will continue to evolve, and if more enzymes of the Benson-Calvin cycle will also acquire similar CP12-like disordered extensions, possibly meaning that the CP12 protein will become redundant. However, CP12 seems to be a part of numerous other processes in photosynthetic organisms (Singh et al., 2008; Howard et al., 2011a,c; Stanley et al., 2013), so it is unlikely to disappear completely from higher plants genomes in the future.

## Rubisco activase

**Figure 2:**
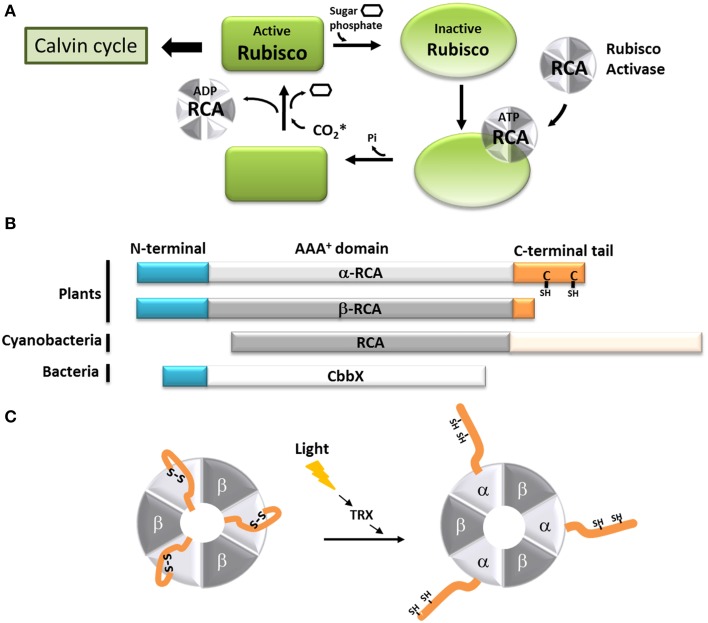
**Model of the function of the C-terminal extension of the α-Rubisco activase. (A)** Schematic representation of the Rubisco activase reaction with Rubisco. Only the carbamylated Rubisco is active and can participate to the Calvin-Benson cycle. However, the presence of sugar phosphate in the Rubisco active site prevents is carbamylation. Using ATP, the Rubisco activase facilitates the departure of the inhibiting sugar phosphate and promotes the Rubisco carbamylation. The CO_2_^*^ represents the non-substrate molecule carbamylating Rubisco. **(B)** Schematic organization of the α and β Rubisco activases subunits from plants, β-cyanobacteria and CbbX. **(C)** Schematic representation of the light-dependent redox regulation of the α_3_β_3_ Rubisco activase. When oxidized, the C-terminal extension of the α-RCA subunit bears a disulfide bridge, which places the C-terminal amino acids inside the nucleotide-binding site of the protein, resulting in its inhibition. The disulfide bridge can be reduced by the thioredoxin *f* (TRX), and Rubisco becomes active.

Ribulose-1,5-bisphosphate carboxylase/oxygenase (Rubisco) is the enzyme that catalyzes the formation of two molecules of phosphoglyceric acid using one molecule of ribulose 1,5-bisphosphate (RuBP) and one of carbon dioxide (CO_2_). As the primary CO_2_ acceptor of most photoautotrophic organisms, Rubisco can represent up to half of soluble proteins in higher plants, and is believed to be the most abundant protein on Earth (Ellis, 1979; Losh et al., 2013; Raven, 2013). Rubisco from most photosynthetic organisms, including plants and cyanobacteria, is a very large protein (550 kDa), composed of large (L, 52 kDa) and small (S, 12 kDa) subunits arranged as a L_8_S_8_ hexadecamer. For this enzyme to be active, a lysine residue inside the Rubisco active site (K201 in *Nicotiana tobacum*) must be carbamylated and bind a Mg^2+^ ion (Lorimer et al., 1976; Andersson and Backlund, 2008). The addition of the non-catalytic CO_2_ molecule to the active site is a spontaneous process, but the presence of RuBP or other sugar-phosphate at the active site decreases the carbamylation efficiency of Rubisco and thus its activity (Lorimer et al., 1976; Cleland et al., 1998). The Rubisco activases (RCAs) exhibit ATPase activity and were first characterized for their ability to promote the carbamylation of such RuBP-inhibited Rubisco (Portis et al., 1986). With time, it became clear that the RCAs allowed the CO_2_ to enter the active site of Rubisco by removing the hindering RuBP or its analog, carboxyarabinitol bisphosphate (CABP) (Figure 2A) (Portis et al., 2008). The presence of RCAs allows Rubisco to function at its maximal capacity in sub-optimal CO_2_ concentration that would normally not permit carbamylation *in vivo* (Portis et al., 1986). In higher plants, RCAs, as expected, are mostly present in the parts of plants involved in photosynthesis (Watillon et al., 1993; Liu et al., 1996), and their expression follows a daily cycle that is regulated by external factors like light and temperature (Martino-Catt and Ort, 1992; Watillon et al., 1993; Liu et al., 1996; To et al., 1999).

**Figure 3:**
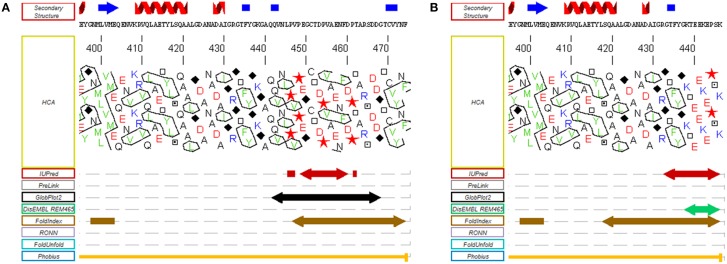
**Disorder predictions of the C-terminal regions of the α and β RCA proteins from *A. thaliana***. MeDor (http://www.vazymolo.org/MeDor/) graphical output of the C-terminal part of the α **(A)** and β **(B)** Rubisco activase isoforms from *A. thaliana*. Predicted secondary structure elements and the HCA plot, are shown above and below the amino acid sequence, respectively. Arrows below the HCA plot correspond to regions of predicted disorder (Lieutaud et al., 2008).

In most organisms from the green lineage, two isoforms of RCA are found: an α isoform of 45–46 kDa and a β isoform of 41–43 kDa (Werneke et al., 1989; Rundle and Zielinski, 1991; To et al., 1999; Gontero and Salvucci, 2014). The only difference between the two RCA isoforms is the presence of a short C-terminal extension (*ca* 30 amino acid residues depending on the species) on the α isoform (Figure 2B). Both the α and β isoforms were found in *Arabidopsis thaliana*, spinach, and rice, although only one RCA gene is present (Werneke et al., 1988; To et al., 1999); in these species, the presence of the two RCA isoforms is the result of alternate splicing (Werneke et al., 1989; Rundle and Zielinski, 1991; To et al., 1999). On the other hand, other species like barley, cotton, maize and tobacco have multiple RCA genes (Rundle and Zielinski, 1991; Qian and Rodermel, 1993; Salvucci et al., 2003; Yin et al., 2010). In most cases, these organisms have separate genes coding for α and β RCAs without alternative splicing (Rundle and Zielinski, 1991), although all the genes identified in tobacco and cucumber appear to only encode the β isoform (Portis, 2003). To the best of our knowledge, the C-terminus of the α and β RCA isoforms were never tested for intrinsic disorder. Using several disorder predictors, including MeDor (Lieutaud et al., 2008) and MFDp2 (Mizianty et al., 2014), we were able to determine that the end of both the C-terminal part of the α and β RCAs (*ca* 50 residues for the α isoform and 20 residues for the β isoform) seem to be intrinsically disordered, including the entire C-terminal extension of the α RCA (Figure 3). The most remarkable features of this disordered tail are the two cysteine residues (C392 and C411 in the *A. thaliana* protein), that are highly conserved among the α RCA isoforms (Zhang and Portis, 1999).

The crystal structure of the tobacco β RCA was recently solved at 3 Å (Stotz et al., 2011), showing that RCA proteins are functional doughnut-shaped hexamers displaying an AAA^+^ fold, as was predicted using other AAA^+^ proteins (ATPases involved in a multitude of processes, Neuwald et al., 1999 as templates Portis, 2003). Interestingly, the last 23 residues of the protein were absent from the structure, indicating that this part of the molecule is very flexible and can adopt different conformations. Moreover, the substrate recognition site of the RCA from the creosote bush, *Larrea tridentata*, was solved at the atomic level (Henderson et al., 2011). Unfortunately, the structural studies were performed only on the β RCA isoform. As the α RCA core is identical, its structure should not be different from the β RCA, but the α RCA was shown to form functional α_*n*_β_*n*_ heteromers rather than α_*n*_ homomers (Crafts-Brandner et al., 1997; Zhang et al., 2001). In the light of these new structural data, we can suppose that α RCA can form heterohexamers α_3_β_3_ (Figure 2C). These structural data also show the presence of three loops containing hydrophilic amino acid residues lining the surface of a central pore. Site-directed mutations introduced in this part of the proteins severely diminished the Rubisco activation and the ATP hydrolysis by the RCA proteins (Stotz et al., 2011), confirming that this region is implicated in the binding of ATP (Salvucci et al., 1993; Li et al., 2006). Based on this information, the model that has been proposed for RCA interaction with Rubisco includes one face of the flat hexamer interacting with the surface of the Rubisco, while an exposed loop of the Rubisco protein could fit into the central hole. Minor conformational changes of this Rubisco loop, allowed by the ATP hydrolysis, would then be transmitted to Rubisco allowing the inhibiting RuBP to be released and Rubisco to be carbamylated (Stotz et al., 2011).

The activity of the α and β RCAs is classically described to be dependent on the ATP:ADP ratio (Zhang and Portis, 1999; Carmo-Silva and Salvucci, 2013), and is extremely sensitive to high temperatures (Portis, 2003; Salvucci, 2004). Moreover, the activity of the α RCA is regulated by light (Mächler and Nösberger, 1980; Perchorowicz et al., 1981). This observation is linked to the action of thioredoxin *f* on the two cysteine residues present on its C-terminal extension (Shen and Ogren, 1992; Zhang and Portis, 1999; Zhang et al., 2001, 2002; Portis, 2003; Wang and Portis, 2006). A site-directed mutagenesis study (Shen and Ogren, 1992) showed that the substitution of only one of the two cysteine residues was enough to abolish the light regulation of α RCA, implicating the involvement of a disulfide bridge. Several studies showed that the mechanism of inhibition involves the blocking of the ATP-binding region by the C-terminal extension upon oxidation. This self-inhibition would be stabilized by strong electrostatic forces between the negatively-charged tail and the positively charged nucleotide site (Shen and Ogren, 1992; Zhang and Portis, 1999; Zhang et al., 2001, 2002; Wang and Portis, 2006; Carmo-Silva and Salvucci, 2013) (Figure 2C). It was also observed that the β RCA, although devoid of regulatory cysteine residues, could be light-regulated in the presence of the α isoform (Zhang and Portis, 1999; Zhang et al., 2001). In the hypothesis that RCAs form α_3_β_3_ heterohexamers, we can assume that the combined bulk of C-terminal extensions efficiently inhibit the whole complex in dark conditions (Figure 2C). The RCA activity can be restored consequently by the reduction of the C-terminal disulfide bridge by the thioredoxin *f*, which occurs upon dark-light transitions (Carmo-Silva and Salvucci, 2013). In this case, the acquisition of a C-terminal tail, originally by alternate splicing, has allowed the RCA protein to fine-tune the activity of Rubisco in function of the light availability in addition to the energetic state of the cell.

In other photosynthetic organisms, the Rubisco activase system is different or works in a different way. In β-cyanobacteria, the RCA protein has the same main domains as plant RCAs, but lacks the N-terminal domain necessary for Rubisco activation found in plants and green algae (Van De Loo and Salvucci, 1996; Li et al., 1999; Stotz et al., 2011; Gontero and Salvucci, 2014; Mueller-Cajar et al., 2014). This could explain why no Rubisco activation has been observed using cyanobacterial RCAs (Li et al., 1999; Pearce, 2006). The latter also possess a very long (180 residues) intrinsically disordered C-terminal extension that seems to target the protein to the carboxysomes (Zarzycki et al., 2013) (Figure 2B). Organisms from the red lineage (α-proteobacteria, rhodophyta, heterokontophyta, etc.) do not have exactly the same Rubisco as the green lineage, and the so-called “Red Rubisco” has a slightly longer large subunit. These organisms do not have RCA genes, but the same Rubisco activase function is carried out by another protein, CbbX (Pearce, 2006; Gontero and Salvucci, 2014) (Figure 2B). The crystal structure of CbbX has recently been solved, showing that this protein is organized in hexamers arranged in a very comparable manner to green RCAs (Mueller-Cajar et al., 2011). It was also suggested that CbbX mechanisms are based on the same principles as the one of RCA, with the C-terminus of the large Rubisco subunit inserted into the central hole of CbbX (Mueller-Cajar et al., 2011). It should be noted that CbbX seems to have an IDR at its C-terminus, but its implications in CbbX activity is yet to be studied.

Rubisco activase is not the only “friendly” protein involved in the regulation of Rubisco, since other proteins are needed during its assembly and folding, including the cpn60 chaperone, which also has a disordered C-terminal tail (Goloubinoff et al., 1989; Cloney et al., 1992; Libich et al., 2013).

## Three transcription factor families (NAC, bZIP and TCP)

Disordered regions are ideal for proteins coordinating regulatory events and as such, transcription factors participating in regulation and signaling functions are enriched in IDRs.

**Figure 4:**
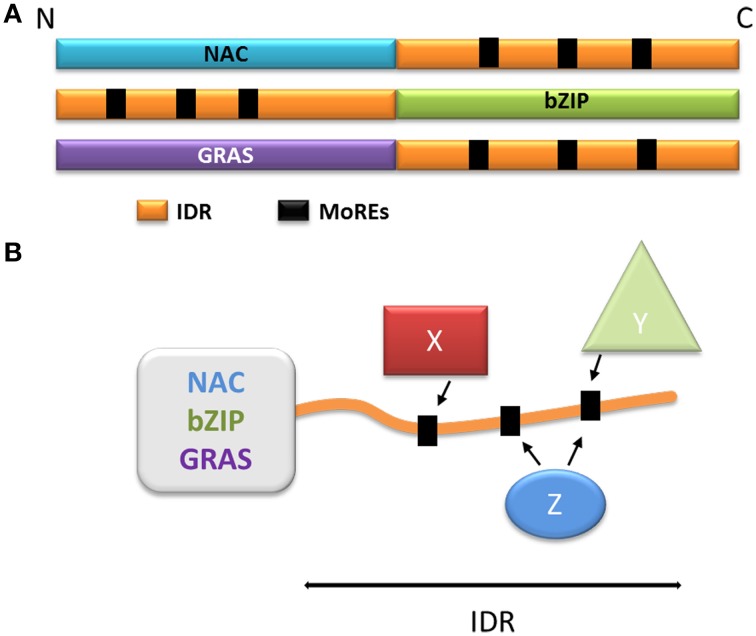
**Model of the function of the disordered extremities of the NAC, bZIP, and GRAS proteins. (A)** Schematic organization of the NAC, bZIP, and GRAS protein families. The disordered parts are schematized in orange, the MoREs are represented as black squares. **(B)** Schematization of the multiple protein-protein interactions involving the disordered extremity. X, Y, and Z represent different protein partners capable of interacting with one or more MoREs and regulate the behavior of the globular domain.

The NAC family (named after No Apical Meristem, ATAF, Cup-Shaped Cotyledon) is one of the largest families of plant-specific transcription factors (Ooka et al., 2003; Olsen et al., 2005; Rushton et al., 2008; Sun et al., 2013). These family members are involved in a very large variety of processes, including plant development (Olsen et al., 2005), biotic and abiotic stress responses (Jensen et al., 2010b; Seo and Park, 2010; Seo et al., 2010) and leaf senescence (Kjaersgaard et al., 2011). The NAC transcription factors usually contain two domains: the N-terminal NAC domain and the C-terminal extremity domain (Figure 4A). The NAC domain is mainly conserved and well-ordered, displaying a typical structure comprising α helices flanking one β strand (Ernst et al., 2004). This domain binds the consensus DNA sequence CGT(GA) (Olsen et al., 2005). The C-terminal domain of the NAC proteins is highly variable within the family; however, some motifs in the C-terminus may display a sub-family-specific conservation (Jensen et al., 2010a). The C-terminal domains composition reveals a very high percentage of hydrophilic (Asp, Glu, Ser, Thr) and proline (Pro) residues, whereas the proportion of hydrophobic and aromatic residues is very low (Olsen et al., 2005; Jensen et al., 2010a). These specificities are typical of IDRs, and the C-terminal domain of some NAC proteins was experimentally characterized as an IDR (Jensen et al., 2010a,b). Despite this IDR feature, some hydrophobic and/or aromatic residues are present in this domain; interestingly, these amino acid residues are often conserved among a subfamily (Jensen et al., 2010a,b; Kjaersgaard et al., 2011). The IDR C-terminal domains of the NAC proteins are predicted to contain MoREs that are conserved in sub-families (Jensen et al., 2010a). It has been experimentally confirmed that these particular residues are very important to the specific function of each sub-group in the NAC family, and are essential to activation mechanisms often involving many different partners (Ooka et al., 2003; Ernst et al., 2004; Taoka et al., 2004; Olsen et al., 2005; Ko et al., 2007; Jensen et al., 2010a) (Figure 4B).

The bZIP (basic Leucine Zipper) transcription factors family is ubiquitous and is one of the largest families of transcription factors in eukaryotes. bZIP transcription factors take part in a multitude of regulatory pathways such as development, metabolism, circadian rhythm and response to stress (Sun et al., 2013). The bZIP proteins are composed of two domains: a C-terminal bZIP domain and a N-terminal activation domain (Figure 4A). The C-terminal bZIP domain gives its name to the family and displays large patches of basic residues and leucine zipper motifs (Ellenberger et al., 1992; Vinson et al., 1993). The leucine zipper regions are organized in α helices and are responsible for the dimerization of the proteins through the formation of a coiled-coil structure (Vinson et al., 1993; Yoon et al., 2006), while the basic regions bind to the DNA molecule (Ellenberger et al., 1992). Interestingly, the basic regions have been described either as fully ordered, very flexible or intrinsically disordered depending on the protein (Bracken et al., 1999; Podust et al., 2001; Moreau et al., 2004; Yoon et al., 2006). When bound to DNA, the basic regions have however been observed as α helices, suggesting that the interaction triggers folding in response to a specific DNA motif (Hollenbeck et al., 2002), illustrating once more the disorder to order transition (induced-fit). The N-terminal regions of bZIP proteins act as regulators (Ang et al., 1998; Sun et al., 2013), and are mostly intrinsically disordered (Campbell et al., 2000; Moreau et al., 2004; Yoon et al., 2006; Sun et al., 2013). These regions typically contain different MoREs, and their flexibility allows the interaction with multiple partners, again by adopting different secondary structures (Ang et al., 1998; Campbell et al., 2000; Oldfield et al., 2005; Yoon et al., 2006) (Figure 4B). Through these activating or inhibiting interactions, transcription of the genes targeted by bZIP proteins is effectively modulated in response to several signals. The N-terminal disordered domain also modulates the activity of bZIP transcription factors through post-translational modifications, and phosphorylation in particular. In plants, bZIP transcription factors can be phosphorylated in response to illumination, which disrupts the interactions between the bZIP proteins and their activating partners (Ciceri et al., 1997; Hardtke et al., 2000). The phosphorylated proteins also have lower affinity for their DNA targets, resulting in a decrease of gene activation (Ciceri et al., 1997; Hardtke et al., 2000). Interestingly, some bZIP proteins also display IDRs in their C-terminal domain. In the case of bZIP28 (initially a transmembrane protein), these IDRs are exposed to the lumen of the endoplasmic reticulum and allow the interaction, through MOREs with BIP, the majority reticulum chaperone. In response to stress, bZIP28 is relocated to the Golgi and the cytoplasmic domain is detached, allowing it to enter the nucleus and to control gene expression (Srivastava et al., 2013, 2014).

A recent study on TCP8, a transcription factor belonging to the TCP [Teosinte branched 1 (tb1), Cycloidea (cyc) and Proliferating Cell Factor (PCF)] family, showed the presence of three IDRs, two of them at the N- and C-terminal extremities (Valsecchi et al., 2013). While the N-terminus binds DNA in an induced-fit mechanism, the C-terminal region is involved in the TCP protein self-association in a coiled-coil structure (Valsecchi et al., 2013). Furthermore, it seems that different transcription factors from the TCP family can interact, modulating the response of different pathways to multiple stimuli (Baier and Latzko, 1975; Viola et al., 2011, 2012; Steiner et al., 2012; Valsecchi et al., 2013).

As illustrated in these examples, the disordered tails of transcription factors have an essential role in modulating their activities through protein-protein interactions with a wide range of activators and inhibitors. Moreover, these extensions are often prone to phosphorylation and constitute another level of regulation. Together, these IDRs form a complex signaling web, turning the transcription factors into hubs and allowing the genes involved in adaptive responses to be finely regulated.

## GRAS family

The GRAS family comprises proteins involved in numerous aspects of plant development and growth. This large family is named after its first members, Gibberellic Acid Insensitive (GAI), Repressor of Gai (RGA) and Scarecrow (SCR), and its members are mostly related to signaling in response to phytohormones [gibberellic acid (GA), auxin, brassinosteroids] and biotic and abiotic stress (Bolle, 2004; Sun et al., 2011). The GRAS family proteins are composed of one variable N-terminal region and a commonly conserved C-terminal GRAS domain (Figure 4A), and are divided into ten subfamilies based on phylogeny (Bolle, 2004; Tian et al., 2004; Lim et al., 2005; Sanchez et al., 2007; Sun et al., 2011). The conserved GRAS domain (*ca* 380 residues depending on the subfamilies) acts as a transcriptional co-activator (Heery et al., 1997) through leucine-rich motifs. GRAS domains typically contain two leucine-rich motifs, which are needed for specific protein-protein interactions(Cui et al., 2007; Vacic et al., 2007; Fode et al., 2008; Hirsch and Oldroyd, 2009; Hirsch et al., 2009; Hou et al., 2010). The GRAS proteins interact with a large number of nuclear proteins, most of which are transcription factors, thereby modulating their target activity (Hirsch and Oldroyd, 2009; Hirsch et al., 2009; Hou et al., 2010; Sun et al., 2012).

In contrast to the highly conserved GRAS domain, the N-terminal domains of the GRAS family proteins display a rich diversity at the sequence level, although the N-terminus is conserved within subfamilies (Sun et al., 2010, 2011, 2012, 2013). Moreover, these N-terminal domains have recently been identified as intrinsically disordered (Sun et al., 2010, 2011, 2012, 2013). Interestingly, patches of repeated hydrophobic and/or aromatic residues are found in the N-terminal region (Triezenberg, 1995; Sun et al., 2011). These patches are arranged in conserved motifs within subfamilies (Triezenberg, 1995; Sun et al., 2011), and are involved in specific multiple protein-protein interactions (Sun et al., 2010, 2012). In the case of the DELLA subfamily which has been intensively studied, the N-terminal domain can interact with the gibberellic acid receptor GIB1, but only when GIB1 has bound its ligand (Murase et al., 2008; Hirano et al., 2010; Sun et al., 2010, 2012). Moreover, each DELLA protein domain (N-terminal and C-terminal domains) can interact with several partners, making these proteins a hub at the center of the gibberellic acid response pathway. Other examples of GRAS proteins are important in other regulatory pathways, although subfamilies are always specialized in a precise type of stimulus (phytohormones, biotic and abiotic stress, etc…) (Sun et al., 2010, 2011, 2012, 2013).

A common feature of the GRAS proteins is their ability to acquire a structure when bound to a partner, unlike the fuzzy GAPDH/CP12 complex (Mileo et al., 2013). As mentioned above, MoREs are present in GRAS proteins; each one was predicted to occur within the N-terminal domains, and more specifically in the elements conserved within subfamilies, strengthening the idea that these motifs are the key to the specificity of GRAS proteins (Sun et al., 2011, 2012). In the case of the DELLA subfamily, the presence of the MoREs has been verified experimentally (Sun et al., 2010, 2011, 2012). Interestingly, the N-terminal domain of the GRAS proteins is also the target of phosphorylation, which again introduces another way to fine-tune the regulation of these proteins (Fu et al., 2002; Iakoucheva et al., 2004; Hussain et al., 2007; Mittag et al., 2010). Phosphorylation of the N-terminal domain is directly linked to the activity of the GRAS proteins, modulating the affinity of the N-terminus for its partners, and having a direct effect on the GRAS proteins stability through the control of their degradation (Day et al., 2004; Hussain et al., 2005; Itoh et al., 2005; Czikkel and Maxwell, 2007).

When considering the GRAS family as a whole, it is remarkable how conserved the GRAS domains and patterns are, while the N-terminal domains are highly variable. It seems that the addition of a disordered protein segment to the GRAS domain has increased its number of partners, and thus turned it into a signal-integration hub involved in many different pathways. On the other hand, one could consider that the addition of GRAS domains to pre-existing IDPs involved in the phytohormonal and/or stress responses has allowed these IDPs to control, even more directly, the cellular responses by acting on gene expression.

## Cryptochrome

Cryptochromes are a group of proteins in which most members have an intrinsically disordered C-terminal tail that can have a profound impact on their overall function. Together with the photolyases, these proteins belong to the photolyase/cryptochrome family (Lin and Shalitin, 2003; Sancar, 2004; Chaves et al., 2006; Ozturk et al., 2007; Fortunato et al., 2015).

Photolyases are ancient enzymes that use blue light to catalyze the repair of DNA lesions caused by ultraviolet light. Lesions such as cyclobutane pyrimidine dimers (CPD) and pyrimidine-pyrimidone photoproducts are repaired by photolyases CPD and by photolyases 6–4, respectively. Photolyase capacity to use blue light is due to the presence of two chromophores: a photoantenna pterin (5,10-methenyltetrahydrofolateor a-hydroxy-5-deazaflavin) and flavin adenine dinucleotide (FAD). During the DNA repair, the two chromophores cofactors absorb blue photons and initiate splitting of the cyclobutane ring by a mechanism involving reactive radicals (Liu et al., 2011b).

**Figure 5:**
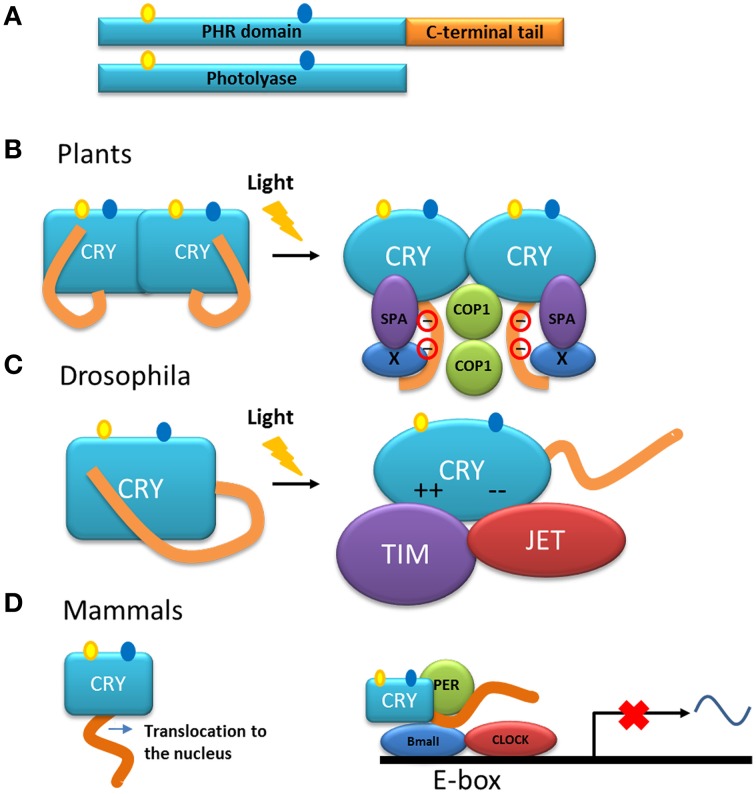
**Models of the function of the C-terminal tail of cryptochromes. (A)** Representation of cryptochrome (CRY) and photolyase. Cryptochromes have a photolyase-homologous region (PHR) and a C-terminal tail. The chromophore molecules of the PHR are shown. **(B)** Model of the action mechanism of cryptochromes from Arabidopsis. After absorption of light, the C-terminal tail is phosphorylated and a change in conformation is triggered in the entire molecule. The C-terminal tail is exposed at the surface of the protein and as a consequence interactions with partner proteins such as COP1 and SPA are induced (Liu et al., 2011a,b). **(C)** In darkness, the C-terminal tail of the cryptochrome from *Drosophila* inhibits the binding of the proteins involved in the circadian rhythm. After illumination, the inhibition by the tail is released and the PHR domain interacts through electrostatic interaction with the protein partners TIM and JET (Green, 2004; Czarna et al., 2013). **(D)** In mammals, cryptochrome is necessary for the translocation of the protein into the nucleus in which it is part of the core of the transcription/translation feedback that controls the circadian clock together with the proteins PER, BMAL, and CLOCK.

Cryptochromes, the other group of proteins in the photolyase/cryptochrome family, have a photolyase homologous region (called PHR) and a C-terminal tail (Figure 5A) (Yu et al., 2010). Cryptochromes are able to absorb blue light in a very similar way to the photolyases. Another group within this family includes DASH-type cryptochromes named after the *Drosophila, Arabidopsis, Synechocystis* and Human. Members of this group are closer to photolyases than to cryptochromes, and are able to repair single-stranded DNA (Chaves et al., 2011) and may also have N-terminal and C-terminal disordered extensions.

In contrast to photolyases, cryptochromes do not have the ability to repair DNA. However, in many organisms, the absorption of photons by the chromophores in the photolyase homologous region of these proteins, induces conformational change (through electron transfer and subsequent phosphorylation), which in turn trigger specialized signaling events through protein-protein interactions (Liu et al., 2011b). It has been shown that the function of cryptochromes resides mainly within their C-terminal tails (Yang et al., 2000; Green, 2004; Chaves et al., 2006, 2011; Yu et al., 2010). Interestingly, this tail is poorly conserved among groups of organisms. In *Arabidopsis*, two cryptochromes are present, CRY1 and CRY2, that have different C-terminal extensions although a DAS motif is found in both (Lin and Shalitin, 2003). The length of the C-terminal tail in cryptochromes of animals, plants and some unicellular organisms varies from 30 to 250 residues and, as mentioned above, is intrinsically disordered. This characteristic has been established by sequence analysis, biochemical methods such as analysis of the sensitivity to protease cleavage, and physical methods such as circular dichroism and nuclear magnetic resonance (NMR)on recombinant C-terminal extensions of both *Arabidopsis* and human cryptochromes (Partch et al., 2005). Comparison of the proteolysis susceptibility between full-length cryptochromes and their C-terminal tail showed that this tail interacts with the photolyase domain, causing it to adopt a tertiary structure. The susceptibility to proteolysis of the C-terminal tail of the CRY1 from *A. thaliana* increases after illumination, which is consistent with a conformational change (Partch et al., 2005). Indeed, the crystal structure of the complete cryptochrome from *Drosophila* confirmed that the C-terminal tail stays in a groove of the photolyase domain and mimics the recognition of photolyases with DNA (Zoltowski et al., 2011; Czarna et al., 2013).

In plants, cryptochromes play a role, together with other photoreceptors, in a variety of functions. In general, the cryptochromes of plants are involved in mechanisms that respond to blue light and their action has been explored in the inhibition of the elongation of hypocotyls, in the photoperiodic induction of flowering, in the circadian clock as in animals, and in other functions (Yu et al., 2010; Chaves et al., 2011; Liu et al., 2011b). These studies have been mainly performed in the model plant *A. thaliana*. Studies using transgenic plants overexpressing the C-terminal tail of CRY1 or CRY2, fused with β-glucuronidase (GUS) showed a constitutive morphogenic phenotype similar to that produced by blue light (Yang et al., 2000), indicating that, in the cryptochrome molecule, the C-terminal tail is responsible for the light-induced function. Moreover, NC80, an 80-residues segment present in the *Arabidopsis* protein, is responsible for the function of the C-terminal tail of CRY2 (Yu et al., 2007). The C-terminal tail of these proteins interacts with other proteins such as COP1 (constitutive photomorphogenic 1) (Wang et al., 2001; Yang et al., 2001), a multifunctional E3 ubiquitin ligase, and SPA1 (suppressor of phytochrome A 1) (Zuo et al., 2011; Liu et al., 2011a). This interaction is part of the initial steps for the light signaling and mechanisms to modulate the developmental process in the plant either by: (1) modulation of gene transcription or (2) suppression of proteolysis of regulators involved in development (i.e., flowering) (Liu et al., 2011a,b). Models have been proposed to explain the mode of action of plant cryptochromes (Lin and Shalitin, 2003; Partch et al., 2005; Yu et al., 2007; Liu et al., 2011a). In general, in these models, the photolyase domain and the C-terminal tail form a closed conformation in the dark. Upon illumination, an open and active conformation is adopted and, in this new conformation, the C-terminal tail is exposed allowing its interaction with other proteins to initiate signaling (Figure 5B). A model of action that includes dimerization and light dependent-phosphorylation that explains the exposure of the C-terminal tail as a result of charge repelling has also been proposed (Figure 5B) (Lin and Shalitin, 2003; Yu et al., 2007).

Although cryptochromes of plants are involved as photoreceptors in the circadian cycle, the molecular role of cryptochromes in relation to this cycle has been more elucidated in *Drosophila*. In this organism, the cryptochrome modulates the central oscillator, or clock, through the light-dependent interaction with the protein Timeless (TIM) (Busza et al., 2004), one of the components of the clock core. This interaction favors the degradation of both TIM and the cryptochrome itself, thus triggering the light/dark cycle each day by synchronization of the clock with the environment. The protein Jetlag (JET), an E3 ligase, also binds to the cryptochrome in a light-dependent manner and is responsible for the ubiquitination and subsequent proteolysis of both the cryptochrome and TIM (Peschel et al., 2009). In this case, and in contrast with the cryptochromes in *Arabidopsis*, the binding of the cryptochrome from *Drosophila* to its partners is performed by the photolyase domain of the protein (Figure 5C), whereas in the dark, the C-terminal tail inhibits this binding determining thus the photosensitivity of the circadian clock (Busza et al., 2004; Green, 2004).

In contrast to their homologs from plants and *Drosophila*, where the disordered C-terminal tail is used for light signaling, mammalian cryptochromes are light–independent transcriptional repressors in the core of the circadian clock (Figure 5D). Mammalian cryptochromes repress transcription processes that are dependent on the protein complex BMAL/CLOCK (Sancar, 2004; Chaves et al., 2011). In the case of these cryptochromes, the function of the C-terminal tail is more complex: (i) it is involved in the nuclear localization of the protein and (ii) with the photolyase domain, it also has a role in the interaction with other components of the clock such as BMAL (Chaves et al., 2006). Interestingly, the C-terminal tail also contributes to the circadian period length, since its phosphorylation affects the level of the protein, either promoting its own degradation in the case of CRY 2 (Harada et al., 2005) or stabilizing the protein as for CRY 1 (Gao et al., 2013).

It has been proposed that cryptochromes have evolved several times independently as an example of convergent evolution (Green, 2004). Only small changes have occurred in the photolyase domain, this part of the protein being conserved among cryptochromes and photolyases. One possible mechanism to explain the acquisition of C-terminal extensions in existing proteins would be through gene fusion (Marsh and Teichmann, 2010). If this mechanism had taken place at the origin of cryptochromes, it would suggest that proteins related to the C-terminal tail of cryptochromes already existed independently and had a function of their own. These independent domains became later associated to a photolyase domain providing them with the capacity to detect light. As mentioned above, the plant cryptochromes C-terminal domain is active and has the information needed to achieve signaling (Yang et al., 2000). During evolution, this protein could have fused with a duplicate of photolyase. In this hypothesis, the addition of the light-dependent photolyase module might be a way to adjust the physiology of the organisms to their environment through light perception. This could therefore be seen as an IDP having acquired a globular extension. Since in plants, a motif (DAS) within the C-terminal tail is conserved, it has been proposed that the ancestral plant cryptochrome emerged from a fusion of a photolyase with a protein containing the DAS motif (Lin and Shalitin, 2003). Another hypothesis that could explain the acquisition of the C-terminal tail in cryptochromes is by gene extension into a non-coding region (Marsh and Teichmann, 2010). The photolyase gene could thus have been extended through junk DNA. Analysis of phylogenetic relationships of gene families in animals showed that extension of an existing gene by “exonization” of a previous non-coding region seems to be an important evolutionary strategy to add a C-terminal disordered extension to proteins (Buljan et al., 2010). The high variability and different functions of the C-terminal tail of cryptochromes among plants and animals are in accordance with this hypothesis. Studies on the origin and evolution of the C-terminal tail of cryptochromes will give insights into the adaptation of organisms to light.

## Conclusion

Within the present review, we tried to demonstrate the central and multiple roles of intrinsically disordered tails carried by certain globular proteins. Describing several examples of proteins displaying IDRs in photosynthetic organisms, we discussed how IDRs impact on both the functions and mechanisms of action of their “host” proteins. The examples of the A_2_B_2_-GAPDH and the α-Rubisco activase isoform show that their C-terminal disordered extensions participate in the light-dependent redox regulation of the photosynthetic metabolism. The cases of the multiple transcription factors with a disordered tail are very similar yet very different. In the few examples listed here, the disordered region plays a major role in the regulation of the DNA-binding domain through protein-protein interactions or post-translational modifications. Their sensitivity to a large number of signals allows the activity of the transcription factors to be modulated according to many factors (one to many), turning these proteins into hubs in a large signaling web. Lastly, the cryptochrome family is a prime example of a disordered extension changing the fundamental function of the initial photolyase into a light-dependent signaling protein, conserving the ability to absorb blue light and repurposing it.

The examples presented here are but a few of the multitude of proteins that have acquired a disordered extension (Uversky, 2013), although most examples do not usually come from the photosynthetic world. We can expect that in the years to come, an increasing number of these proteins will be identified. A great question that remains is how these proteins originated. While in some cases, the addition of an IDR seems to be quite recent like the GapB subunit. In other cases, this addition might be very ancient as in the NAC, bZIP, and GRAS families, in which there are multiple disordered extensions families that may derive from multiple fusion events, or a long succession of duplications followed by diverging evolution of the subfamilies. We hope that the expansion of the IDP field in general and specifically, the one involved in “green” biochemistry, will 1 day answer these questions.

### Conflict of interest statement

The authors declare that the research was conducted in the absence of any commercial or financial relationships that could be construed as a potential conflict of interest.

## References

[B1] Alderson T. R. Markley J. L. (2013). Biophysical characterization of alpha-synuclein and its controversial structure. Intrinsically Disord. Proteins 1, 18–39. 10.4161/idp.26255 24634806 PMC3908606

[B2] Andersson I. Backlund A. (2008). Structure and function of Rubisco. Plant Physiol. Biochem. 46, 275–291. 10.1016/j.plaphy.2008.01.001 18294858

[B3] Ang L. H. Chattopadhyay S. Wei N. Oyama T. Okada K. Batschauer A.. (1998). Molecular interaction between COP1 and HY5 defines a regulatory switch for light control of *Arabidopsis* development. Mol. Cell 1, 213–222. 10.1016/S1097-2765(00)80022-2 9659918

[B4] Arabidopsis Genome Initiative. (2000). Analysis of the genome sequence of the flowering plant *Arabidopsis thaliana*. Nature 408, 796–815. 10.1038/35048692 11130711

[B5] Avilan L. Gontero B. Lebreton S. Ricard J. (1997). Memory and imprinting effects in multienzyme complexes–I. Isolation, dissociation, and reassociation of a phosphoribulokinase-glyceraldehyde-3-phosphate dehydrogenase complex from *Chlamydomonas reinhardtii* chloroplasts. Eur. J. Biochem. 246, 78–84. 10.1111/j.1432-1033.1997.00078.x 9210468

[B6] Avilan L. Lebreton S. Gontero B. (2000). Thioredoxin activation of phosphoribulokinase in a bi-enzyme complex from *Chlamydomonas reinhardtii* chloroplasts. J. Biol. Chem. 275, 9447–9451. 10.1074/jbc.275.13.9447 10734091

[B7] Avilan L. Puppo C. Erales J. Woudstra M. Lebrun R. Gontero B. (2012). CP12 residues involved in the formation and regulation of the glyceraldehyde-3-phosphate dehydrogenase-CP12-phosphoribulokinase complex in *Chlamydomonas reinhardtii*. Mol. Biosyst. 8, 2994–3002. 10.1039/c2mb25244a 22955105

[B8] Baalmann E. Backhausen J. E. Kitzmann C. Scheibe R. (1994). Regulation of NADP-dependent glyceraldehyde 3-phosphate dehydrogenase activity in spinach chloroplast. Botanica Acta 107, 313–320. 10.1111/j.1438-8677.1994.tb00801.x

[B9] Baalmann E. Backhausen J. E. Rak C. Vetter S. Scheibe R. (1995). Reductive modification and nonreductive activation of purified spinach chloroplast NADP-dependent glyceraldehyde-3-phosphate dehydrogenase. Arch. Biochem. Biophys. 324, 201–208. 10.1006/abbi.1995.0031 8554310

[B10] Baalmann E. Scheibe R. Cerff R. Martin W. (1996). Functional studies of chloroplast glyceraldehyde-3-phosphate dehydrogenase subunits A and B expressed in *Escherichia coli*: formation of highly active A_4_ and B_4_ homotetramers and evidence that aggregation of the B_4_ complex is mediated by the B subunit carboxy terminus. Plant Mol. Biol. 32, 505–513. 10.1007/BF00019102 8980499

[B11] Babu M. M. Van Der Lee R. De Groot N. S. Gsponer J. (2011). Intrinsically disordered proteins: regulation and disease. Curr. Opin. Struct. Biol. 21, 432–440. 10.1016/j.sbi.2011.03.011 21514144

[B12] Baier D. Latzko E. (1975). Properties and regulation of C-1-fructose-1,6-diphosphatase from spinach chloroplasts. Biochim. Biophys. Acta 396, 141–148. 10.1016/0005-2728(75)90197-8 238625

[B13] Bolle C. (2004). The role of GRAS proteins in plant signal transduction and development. Planta 218, 683–692. 10.1007/s00425-004-1203-z 14760535

[B14] Bracken C. Carr P. A. Cavanagh J. Palmer A. G. III. (1999). Temperature dependence of intramolecular dynamics of the basic leucine zipper of GCN4: implications for the entropy of association with DNA. J. Mol. Biol. 285, 2133–2146. 10.1006/jmbi.1998.2429 9925790

[B15] Breydo L. Uversky V. N. (2011). Role of metal ions in aggregation of intrinsically disordered proteins in neurodegenerative diseases. Metallomics 3, 1163–1180. 10.1039/c1mt00106j 21869995

[B16] Breydo L. Wu J. W. Uversky V. N. (2012). Alpha-synuclein misfolding and Parkinson's disease. Biochim. Biophys. Acta 1822, 261–285. 10.1016/j.bbadis.2011.10.002 22024360

[B17] Brinkmann H. Cerff R. Salomon M. Soll J. (1989). Cloning and sequence analysis of cDNAs encoding the cytosolic precursors of subunits GapA and GapB of chloroplast glyceraldehyde-3- phosphate dehydrogenase from pea and spinach. Plant Mol. Biol. 13, 81–94. 10.1007/BF00027337 2562762

[B18] Buljan M. Frankish A. Bateman A. (2010). Quantifying the mechanisms of domain gain in animal proteins. Genome Biol. 11:R74. 10.1186/gb-2010-11-7-r74 20633280 PMC2926785

[B19] Busza A. Emery-Le M. Rosbash M. Emery P. (2004). Roles of the two *Drosophila* CRYPTOCHROME structural domains in circadian photoreception. Science 304, 1503–1506. 10.1126/science.1096973 15178801

[B20] Campbell K. M. Terrell A. R. Laybourn P. J. Lumb K. J. (2000). Intrinsic structural disorder of the C-terminal activation domain from the bZIP transcription factor Fos. Biochemistry 39, 2708–2713. 10.1021/bi9923555 10704222

[B21] Carmo-Silva A. E. Salvucci M. E. (2013). The regulatory properties of Rubisco activase differ among species and affect photosynthetic induction during light transitions. Plant Physiol. 161, 1645–1655. 10.1104/pp.112.213348 23417088 PMC3613445

[B22] Castillo P. Cetina A. F. Mendez-Tenorio A. Espinoza-Fonseca L. M. Barron B. L. (2014). Papillomavirus binding factor (PBF) is an intrinsically disordered protein with potential participation in osteosarcoma genesis, in silico evidence. Theor. Biol. Med. Model. 11:51. 10.1186/1742-4682-11-51 25471943 PMC4265421

[B23] Cerff R. (1979). Quaternary structure of higher plant glyceraldehyde-3-phosphate dehydrogenases. Eur. J. Biochem. 94, 243–247. 10.1111/j.1432-1033.1979.tb12891.x 35350

[B24] Chakrabortee S. Tripathi R. Watson M. Schierle G. S. Kurniawan D. P. Kaminski C. F.. (2012). Intrinsically disordered proteins as molecular shields. Mol. Biosyst. 8, 210–219. 10.1039/C1MB05263B 21909508 PMC5365143

[B25] Chaves I. Pokorny R. Byrdin M. Hoang N. Ritz T. Brettel K.. (2011). The cryptochromes: blue light photoreceptors in plants and animals. Annu. Rev. Plant Biol. 62, 335–364. 10.1146/annurev-arplant-042110-103759 21526969

[B26] Chaves I. Yagita K. Barnhoorn S. Okamura H. Van Der Horst G. T. Tamanini F. (2006). Functional evolution of the photolyase/cryptochrome protein family: importance of the C-terminus of mammalian CRY1 for circadian core oscillator performance. Mol. Cell. Biol. 26, 1743–1753. 10.1128/MCB.26.5.1743-1753.2006 16478995 PMC1430250

[B27] Chouard T. (2011). Structural biology: breaking the protein rules. Nature 471, 151–153. 10.1038/471151a 21390105

[B28] Ciceri P. Gianazza E. Lazzari B. Lippoli G. Genga A. Hoscheck G.. (1997). Phosphorylation of Opaque2 changes diurnally and impacts its DNA binding activity. Plant Cell 9, 97–108. 10.1105/tpc.9.1.97 9014367 PMC156904

[B29] Cleland W. W. Andrews T. J. Gutteridge S. Hartman F. C. Lorimer G. H. (1998). Mechanism of Rubisco: the carbamate as general base. Chem. Rev. 98, 549–562. 10.1021/cr970010r 11848907

[B30] Cloney L. P. Bekkaoui D. R. Wood M. G. Hemmingsen S. M. (1992). Assessment of plant chaperonin-60 gene function in Escherichia coli. J. Biol. Chem. 267, 23333–23336. 1358883

[B31] Crafts-Brandner S. J. Van De Loo F. J. Salvucci M. E. (1997). The two forms of ribulose-1,5-bisphosphate carboxylase/oxygenase activase differ in sensitivity to elevated temperature. Plant Physiol. 114, 439–444. 12223718 10.1104/pp.114.2.439 PMC158323

[B32] Cui H. Levesque M. P. Vernoux T. Jung J. W. Paquette A. J. Gallagher K. L.. (2007). An evolutionarily conserved mechanism delimiting SHR movement defines a single layer of endodermis in plants. Science 316, 421–425. 10.1126/science.1139531 17446396

[B33] Czarna A. Berndt A. Singh H. R. Grudziecki A. Ladurner A. G. Timinszky G.. (2013). Structures of *Drosophila* cryptochrome and mouse cryptochrome1 provide insight into circadian function. Cell 153, 1394–1405. 10.1016/j.cell.2013.05.011 23746849

[B34] Czikkel B. E. Maxwell D. P. (2007). NtGRAS1, a novel stress-induced member of the GRAS family in tobacco, localizes to the nucleus. J. Plant Physiol. 164, 1220–1230. 10.1016/j.jplph.2006.07.010 17007961

[B35] Day R. B. Tanabe S. Koshioka M. Mitsui T. Itoh H. Ueguchi-Tanaka M.. (2004). Two rice GRAS family genes responsive to N -acetylchitooligosaccharide elicitor are induced by phytoactive gibberellins: evidence for cross-talk between elicitor and gibberellin signaling in rice cells. Plant Mol. Biol. 54, 261–272. 10.1023/B:PLAN.0000028792.72343.ee 15159627

[B36] Dunker A. K. Brown C. J. Lawson J. D. Iakoucheva L. M. Obradovic Z. (2002). Intrinsic disorder and protein function. Biochemistry 41, 6573–6582. 10.1021/bi012159+ 12022860

[B37] Dunker A. K. Cortese M. S. Romero P. Iakoucheva L. M. Uversky V. N. (2005). Flexible nets. The roles of intrinsic disorder in protein interaction networks. FEBS J. 272, 5129–5148. 10.1111/j.1742-4658.2005.04948.x 16218947

[B38] Dunker A. K. Lawson J. D. Brown C. J. Williams R. M. Romero P. Oh J. S.. (2001). Intrinsically disordered protein. J. Mol. Graph. Model. 19, 26–59. 10.1016/S1093-3263(00)00138-8 11381529

[B39] Dunker A. K. Oldfield C. J. Meng J. Romero P. Yang J. Y. Chen J. W.. (2008a). The unfoldomics decade: an update on intrinsically disordered proteins. BMC Genomics 9(Suppl. 2):S1. 10.1186/1471-2164-9-S2-S1 18831774 PMC2559873

[B40] Dunker A. K. Silman I. Uversky V. N. Sussman J. L. (2008b). Function and structure of inherently disordered proteins. Curr. Opin. Struct. Biol. 18, 756–764. 10.1016/j.sbi.2008.10.002 18952168

[B41] Ellenberger T. E. Brandl C. J. Struhl K. Harrison S. C. (1992). The GCN4 basic region leucine zipper binds DNA as a dimer of uninterrupted alpha helices: crystal structure of the protein-DNA complex. Cell 71, 1223–1237. 10.1016/S0092-8674(05)80070-4 1473154

[B42] Ellis R. J. (1979). The most abundant protein in the world. Trends Biochem. Sci. 4, 241–244. 10.1016/0968-0004(79)90212-3

[B43] Erales J. Avilan L. Lebreton S. Gontero B. (2008). Exploring CP12 binding proteins revealed aldolase as a new partner for the phosphoribulokinase/glyceraldehyde 3-phosphate dehydrogenase/CP12 complex - purification and kinetic characterization of this enzyme from *Chlamydomonas reinhardtii*. FEBS J. 275, 1248–1259. 10.1111/j.1742-4658.2008.06284.x 18266760

[B44] Erales J. Mekhalfi M. Woudstra M. Gontero B. (2011). Molecular mechanism of NADPH-glyceraldehyde-3-phosphate dehydrogenase regulation through the C-terminus of CP12 in *Chlamydomonas reinhardtii*. Biochemistry 50, 2881–2888. 10.1021/bi1020259 21366264

[B45] Ernst H. A. Olsen A. N. Larsen S. Lo Leggio L. (2004). Structure of the conserved domain of ANAC, a member of the NAC family of transcription factors. EMBO Rep. 5, 297–303. 10.1038/sj.embor.7400093 15083810 PMC1299004

[B46] Falini G. Fermani S. Ripamonti A. Sabatino P. Sparla F. Pupillo P.. (2003). Dual coenzyme specificity of photosynthetic glyceraldehyde 3-phosphate dehydrogenase interpreted by the crystal structure of A_4_ isoform complexed with NAD. Biochemistry 42, 4631–4639. 10.1021/bi0272149 12705826

[B47] Fermani S. Ripamonti A. Sabatino P. Zanotti G. Scagliarini S. Sparla F.. (2001). Crystal structure of the non-regulatory A_4_ isoform of spinach chloroplast glyceraldehyde-3-phosphate dehydrogenase complexed with NADP. J. Mol. Biol. 314, 527–542. 10.1006/jmbi.2001.5172 11846565

[B48] Fermani S. Sparla F. Falini G. Martelli P. L. Casadio R. Pupillo P.. (2007). Molecular mechanism of thioredoxin regulation in photosynthetic A_2_B_2_-glyceraldehyde-3-phosphate dehydrogenase. Proc. Natl. Acad. Sci. U.S.A. 104, 11109–11114. 10.1073/pnas.0611636104 17573533 PMC1904167

[B49] Fermani S. Trivelli X. Sparla F. Thumiger A. Calvaresi M. Marri L.. (2012). Conformational selection and folding-upon-binding of the intrinsically disordered protein CP12 regulate photosynthetic enzymes assembly. J. Biol. Chem. 287, 21372–21383. 10.1074/jbc.M112.350355 22514274 PMC3375559

[B50] Fersht A. (1998). Structure and Mechanism in Protein Science: a Guide to Enzyme Catalysis and Protein Folding. New York: W. H. Freeman and Company.

[B51] Fode B. Siemsen T. Thurow C. Weigel R. Gatz C. (2008). The *Arabidopsis* GRAS protein SCL14 interacts with class II TGA transcription factors and is essential for the activation of stress-inducible promoters. Plant Cell 20, 3122–3135. 10.1105/tpc.108.058974 18984675 PMC2613660

[B52] Fortunato A. E. Annunziata R. Jaubert M. Bouly J. P. Falciatore A. (2015). Dealing with light: the widespread and multitasking cryptochrome/photolyase family in photosynthetic organisms. J. Plant Physiol. 172, 42–54. 10.1016/j.jplph.2014.06.011 25087009

[B53] Fu X. Richards D. E. Ait-Ali T. Hynes L. W. Ougham H. Peng J.. (2002). Gibberellin-mediated proteasome-dependent degradation of the barley DELLA protein SLN1 repressor. Plant Cell 14, 3191–3200. 10.1105/tpc.006197 12468736 PMC151211

[B54] Fuxreiter M. Simon I. Friedrich P. Tompa P. (2004). Preformed structural elements feature in partner recognition by intrinsically unstructured proteins. J. Mol. Biol. 338, 1015–1026. 10.1016/j.jmb.2004.03.017 15111064

[B55] Fuxreiter M. Tompa P. (2012). Fuzzy complexes: a more stochastic view of protein function. Adv. Exp. Med. Biol. 725, 1–14. 10.1007/978-1-4614-0659-4_1 22399315

[B56] Fuxreiter M. Tompa P. Simon I. (2007). Local structural disorder imparts plasticity on linear motifs. Bioinformatics 23, 950–956. 10.1093/bioinformatics/btm035 17387114

[B57] Gao P. Yoo S. H. Lee K. J. Rosensweig C. Takahashi J. S. Chen B. P.. (2013). Phosphorylation of the cryptochrome 1 C-terminal tail regulates circadian period length. J. Biol. Chem. 288, 35277–35286. 10.1074/jbc.M113.509604 24158435 PMC3853276

[B58] Gardebien F. Thangudu R. R. Gontero B. Offmann B. (2006). Construction of a 3D model of CP12, a protein linker. J. Mol. Graph. Model. 25, 186–195. 10.1016/j.jmgm.2005.12.003 16427344

[B59] Gavin A. C. Aloy P. Grandi P. Krause R. Boesche M. Marzioch M.. (2006). Proteome survey reveals modularity of the yeast cell machinery. Nature 440, 631–636. 10.1038/nature04532 16429126

[B60] Gavin A. C. Bosche M. Krause R. Grandi P. Marzioch M. Bauer A.. (2002). Functional organization of the yeast proteome by systematic analysis of protein complexes. Nature 415, 141–147. 10.1038/415141a 11805826

[B61] Goloubinoff P. Christeller J. T. Gatenby A. A. Lorimer G. H. (1989). Reconstitution of active dimeric ribulose bisphosphate carboxylase from an unfoleded state depends on two chaperonin proteins and Mg-ATP. Nature 342, 884–889. 10.1038/342884a0 10532860

[B62] Gontero B. Maberly S. C. (2012). An intrinsically disordered protein, CP12: jack of all trades and master of the Calvin cycle. Biochem. Soc. Trans. 40, 995–999. 10.1042/BST20120097 22988853

[B63] Gontero B. Salvucci M. E. (2014). Regulation of photosynthetic carbon metabolism in aquatic and terrestrial organisms by Rubisco activase, redox-modulation and CP12. Aquat. Bot. 118, 14–23. 10.1016/j.aquabot.2014.05.011

[B64] Graciet E. Gans P. Wedel N. Lebreton S. Camadro J. M. Gontero B. (2003a). The small protein CP12: a protein linker for supramolecular assembly. Biochemistry 42, 8163–8170. 10.1021/bi034474x 12846565

[B65] Graciet E. Lebreton S. Camadro J. M. Gontero B. (2003b). Characterization of native and recombinant A_4_ glyceraldehyde 3- phosphate dehydrogenase. Eur. J. Biochem. 270, 129–136. 10.1046/j.1432-1033.2003.03372.x 12492483

[B66] Green C. B. (2004). Cryptochromes: tail-ored for distinct functions. Curr. Biol. 14, R847–R849. 10.1016/j.cub.2004.09.040 15458665

[B67] Groben R. Kaloudas D. Raines C. A. Offmann B. Maberly S. C. Gontero B. (2010). Comparative sequence analysis of CP12, a small protein involved in the formation of a Calvin cycle complex in photosynthetic organisms. Photosynth. Res. 103, 183–194. 10.1007/s11120-010-9542-z 20224939

[B68] Harada Y. Sakai M. Kurabayashi N. Hirota T. Fukada Y. (2005). Ser-557-phosphorylated mCRY2 is degraded upon synergistic phosphorylation by glycogen synthase kinase-3 beta. J. Biol. Chem. 280, 31714–31721. 10.1074/jbc.M506225200 15980066

[B69] Hardtke C. S. Gohda K. Osterlund M. T. Oyama T. Okada K. Deng X. W. (2000). HY5 stability and activity in *Arabidopsis* is regulated by phosphorylation in its COP1 binding domain. EMBO J. 19, 4997–5006. 10.1093/emboj/19.18.4997 10990463 PMC314229

[B70] Haynes C. Oldfield C. J. Ji F. Klitgord N. Cusick M. E. Radivojac P.. (2006). Intrinsic disorder is a common feature of hub proteins from four eukaryotic interactomes. PLoS Comput. Biol. 2:e100. 10.1371/journal.pcbi.0020100 16884331 PMC1526461

[B71] Hazy E. Bokor M. Kalmar L. Gelencser A. Kamasa P. Han K. H.. (2011). Distinct hydration properties of wild-type and familial point mutant A53T of alpha-synuclein associated with Parkinson's disease. Biophys. J. 101, 2260–2266. 10.1016/j.bpj.2011.08.052 22067166 PMC3207174

[B72] Hazy E. Tompa P. (2009). Limitations of induced folding in molecular recognition by intrinsically disordered proteins. Chemphyschem 10, 1415–1419. 10.1002/cphc.200900205 19462392

[B73] Heery D. M. Kalkhoven E. Hoare S. Parker M. G. (1997). A signature motif in transcriptional co-activators mediates binding to nuclear receptors. Nature 387, 733–736. 10.1038/42750 9192902

[B74] Henderson J. N. Kuriata A. M. Fromme R. Salvucci M. E. Wachter R. M. (2011). Atomic resolution x-ray structure of the substrate recognition domain of higher plant ribulose-bisphosphate carboxylase/oxygenase (Rubisco) activase. J. Biol. Chem. 286, 35683–35688. 10.1074/jbc.C111.289595 21880724 PMC3195603

[B75] Hildebrandt T. Knuesting J. Berndt C. Morgan B. Scheibe R. (2015). Cytosolic thiol switches regulating basic cellular functions: GAPDH as an information hub? Biol. Chem. 396, 523–537. 10.1515/hsz-2014-0295 25581756

[B76] Hirano K. Asano K. Tsuji H. Kawamura M. Mori H. Kitano H.. (2010). Characterization of the molecular mechanism underlying gibberellin perception complex formation in rice. Plant Cell 22, 2680–2696. 10.1105/tpc.110.075549 20716699 PMC2947161

[B77] Hirsch S. Kim J. Munoz A. Heckmann A. B. Downie J. A. Oldroyd G. E. (2009). GRAS proteins form a DNA binding complex to induce gene expression during nodulation signaling in *Medicago truncatula*. Plant Cell 21, 545–557. 10.1105/tpc.108.064501 19252081 PMC2660633

[B78] Hirsch S. Oldroyd G. E. (2009). GRAS-domain transcription factors that regulate plant development. Plant Signal. Behav. 4, 698–700. 10.4161/psb.4.8.9176 19820314 PMC2801379

[B79] Hollenbeck J. J. Mcclain D. L. Oakley M. G. (2002). The role of helix stabilizing residues in GCN4 basic region folding and DNA binding. Protein Sci. 11, 2740–2747. 10.1110/ps.0211102 12381856 PMC2373721

[B80] Hou X. Lee L. Y. Xia K. Yan Y. Yu H. (2010). DELLAs modulate jasmonate signaling via competitive binding to JAZs. Dev. Cell 19, 884–894. 10.1016/j.devcel.2010.10.024 21145503

[B81] Howard T. P. Fryer M. J. Singh P. Metodiev M. Lytovchenko A. Obata T.. (2011a). Antisense suppression of the small chloroplast protein CP12 in tobacco alters carbon partitioning and severely restricts growth. Plant Physiol. 157, 620–631. 10.1104/pp.111.183806 21865489 PMC3192581

[B82] Howard T. P. Lloyd J. C. Raines C. A. (2011b). Inter-species variation in the oligomeric states of the higher plant Calvin cycle enzymes glyceraldehyde-3-phosphate dehydrogenase and phosphoribulokinase. J. Exp. Bot. 62, 3799–3805. 10.1093/jxb/err057 21498632 PMC3134340

[B83] Howard T. P. Upton G. J. Lloyd J. C. Raines C. A. (2011c). Antisense suppression of the small chloroplast protein CP12 in tobacco: a transcriptional viewpoint. Plant Signal. Behav. 6, 2026–2030. 10.4161/psb.6.12.18055 22112458 PMC3337198

[B84] Hussain A. Cao D. Cheng H. Wen Z. Peng J. (2005). Identification of the conserved serine/threonine residues important for gibberellin-sensitivity of *Arabidopsis* RGL2 protein. Plant J. 44, 88–99. 10.1111/j.1365-313X.2005.02512.x 16167898

[B85] Hussain A. Cao D. Peng J. (2007). Identification of conserved tyrosine residues important for gibberellin sensitivity of *Arabidopsis* RGL2 protein. Planta 226, 475–483. 10.1007/s00425-007-0497-z 17333251

[B86] Iakoucheva L. M. Radivojac P. Brown C. J. O'connor T. R. Sikes J. G. Obradovic Z.. (2004). The importance of intrinsic disorder for protein phosphorylation. Nucleic Acids Res. 32, 1037–1049. 10.1093/nar/gkh253 14960716 PMC373391

[B87] Itoh H. Sasaki A. Ueguchi-Tanaka M. Ishiyama K. Kobayashi M. Hasegawa Y.. (2005). Dissection of the phosphorylation of rice DELLA protein, SLENDER RICE1. Plant Cell Physiol. 46, 1392–1399. 10.1093/pcp/pci152 15979983

[B88] Jeffery C. J. (1999). Moonlighting proteins. Trends Biochem. Sci. 24, 8–11. 10.1016/S0968-0004(98)01335-8 10087914

[B89] Jensen M. K. Kjaersgaard T. Nielsen M. M. Galberg P. Petersen K. O'shea C.. (2010a). The *Arabidopsis thaliana* NAC transcription factor family: structure-function relationships and determinants of ANAC019 stress signalling. Biochem. J. 426, 183–196. 10.1042/BJ20091234 19995345

[B90] Jensen M. K. Kjaersgaard T. Petersen K. Skriver K. (2010b). NAC genes: time-specific regulators of hormonal signaling in *Arabidopsis*. Plant Signal. Behav. 5, 907–910. 10.4161/psb.5.7.12099 20484991 PMC3014545

[B91] Kaaki W. Woudstra M. Gontero B. Halgand F. (2013). Exploration of CP12 conformational changes and of quaternary structural properties using electrospray ionization traveling wave ion mobility mass spectrometry. Rapid. Commun. Mass Spectrom. 27, 179–186. 10.1002/rcm.6442 23239332

[B92] Karagoz G. E. Rudiger S. G. (2015). Hsp90 interaction with clients. Trends Biochem. Sci. 40, 117–125. 10.1016/j.tibs.2014.12.002 25579468

[B93] Kirschner K. Bisswanger H. (1976). Multifunctional proteins. Annu. Rev. Biochem. 45, 143–166. 10.1146/annurev.bi.45.070176.001043 786148

[B94] Kjaersgaard T. Jensen M. K. Christiansen M. W. Gregersen P. Kragelund B. B. Skriver K. (2011). Senescence-associated barley NAC (NAM, ATAF1,2, CUC) transcription factor interacts with radical-induced cell death 1 through a disordered regulatory domain. J. Biol. Chem. 286, 35418–35429. 10.1074/jbc.M111.247221 21856750 PMC3195629

[B95] Ko J. H. Yang S. H. Park A. H. Lerouxel O. Han K. H. (2007). ANAC012, a member of the plant-specific NAC transcription factor family, negatively regulates xylary fiber development in *Arabidopsis thaliana*. Plant J. 50, 1035–1048. 10.1111/j.1365-313X.2007.03109.x 17565617

[B96] Koshland D. E. Jr. Nemethy G. Filmer D. (1966). Comparison of experimental binding data and theoretical models in proteins containing subunits. Biochemistry 5, 365–385. 10.1021/bi00865a047 5938952

[B97] Kovacech B. Novak M. (2010). Tau truncation is a productive posttranslational modification of neurofibrillary degeneration in Alzheimer's disease. Curr. Alzheimer Res. 7, 708–716. 10.2174/156720510793611556 20678071

[B98] Kragelund B. B. Jensen M. K. Skriver K. (2012). Order by disorder in plant signaling. Trends Plant Sci. 17, 625–632. 10.1016/j.tplants.2012.06.010 22819467

[B99] Krisko A. Smole Z. Debret G. Nikolic N. Radman M. (2010). Unstructured hydrophilic sequences in prokaryotic proteomes correlate with dehydration tolerance and host association. J. Mol. Biol. 402, 775–782. 10.1016/j.jmb.2010.08.012 20709076

[B100] Lebreton S. Gontero B. Avilan L. Ricard J. (1997). Memory and imprinting effects in multienzyme complexes–II. Kinetics of the bienzyme complex from *Chlamydomonas reinhardtii* and hysteretic activation of chloroplast oxidized phosphoribulokinase. Eur. J. Biochem. 246, 85–91. 10.1111/j.1432-1033.1997.t01-2-00085.x 9210469

[B101] Li A. D. Anderson L. E. (1997). Expression and characterization of pea chloroplastic glyceraldehyde-3-phosphate dehydrogenase composed of only the B-subunit. Plant Physiol. 115, 1201–1209. 10.1104/pp.115.3.1201 9390445 PMC158585

[B102] Li C. Wang D. Portis A. R. Jr. (2006). Identification of critical arginine residues in the functioning of Rubisco activase. Arch. Biochem. Biophys. 450, 176–182. 10.1016/j.abb.2006.04.002 16712773

[B103] Li L. A. Zianni M. R. Tabita F. R. (1999). Inactivation of the monocistronic *rca* gene in *Anabaena variabilis* suggests a physiological ribulose bisphosphate carboxylase/oxygenase activase-like function in heterocystous cyanobacteria. Plant Mol. Biol. 40, 467–478. 10.1023/A:1006251808625 10437830

[B104] Libich D. S. Fawzi N. L. Ying J. Clore G. M. (2013). Probing the transient dark state of substrate binding to GroEL by relaxation-based solution NMR. Proc. Natl. Acad. Sci. U.S.A. 110, 11361–11366. 10.1073/pnas.1305715110 23798407 PMC3710837

[B105] Lieutaud P. Canard B. Longhi S. (2008). MeDor: a metaserver for predicting protein disorder. BMC Genomics 9(Suppl. 2):S25. 10.1186/1471-2164-9-S2-S25 18831791 PMC2559890

[B106] Lim J. Jung J. W. Lim C. E. Lee M. H. Kim B. J. Kim M.. (2005). Conservation and diversification of SCARECROW in maize. Plant Mol. Biol. 59, 619–630. 10.1007/s11103-005-0578-y 16244911 PMC1475827

[B107] Lin C. Shalitin D. (2003). Cryptochrome structure and signal transduction. Annu. Rev. Plant Biol. 54, 469–496. 10.1146/annurev.arplant.54.110901.160901 14503000

[B108] Liu B. Zuo Z. Liu H. Liu X. Lin C. (2011a). *Arabidopsis* cryptochrome 1 interacts with SPA1 to suppress COP1 activity in response to blue light. Genes Dev. 25, 1029–1034. 10.1101/gad.2025011 21511871 PMC3093118

[B109] Liu H. Liu B. Zhao C. Pepper M. Lin C. (2011b). The action mechanisms of plant cryptochromes. Trends Plant Sci. 16, 684–691. 10.1016/j.tplants.2011.09.002 21983106 PMC3277817

[B110] Liu Z. Taub C. C. Mcclung C. R. (1996). Identification of an *Arabidopsis thaliana* ribulose-1,5-bisphosphate carboxylase/oxygenase activase (RCA) minimal promoter regulated by light and the circadian clock. Plant Physiol. 112, 43–51. 10.1104/pp.112.1.43 8819320 PMC157921

[B111] Lopez-Calcagno P. E. Howard T. P. Raines C. A. (2014). The CP12 protein family: a thioredoxin-mediated metabolic switch? Front. Plant. Sci. 5:9. 10.3389/fpls.2014.00009 24523724 PMC3906501

[B112] Lorimer G. H. Badger M. R. Andrews T. J. (1976). The activation of ribulose-1,5-bisphosphate carboxylase by carbon dioxide and magnesium ions. Equilibria, kinetics, a suggested mechanism, and physiological implications. Biochemistry 15, 529–536. 10.1021/bi00648a012 3199

[B113] Losh J. L. Young J. N. Morel F. M. (2013). Rubisco is a small fraction of total protein in marine phytoplankton. New Phytol. 198, 52–58. 10.1111/nph.12143 23343368

[B114] Mächler F. Nösberger J. (1980). Regulation of ribulose bisphosphate carboxylase activity in intact wheat leaves by light, CO_2_, and temperature. J. Exp. Bot. 31, 1485–1491. 10.1093/jxb/31.6.1485

[B115] Marri L. Sparla F. Pupillo P. Trost P. (2005). Co-ordinated gene expression of photosynthetic glyceraldehyde-3-phosphate dehydrogenase, phosphoribulokinase, and CP12 in *Arabidopsis thaliana*. J. Exp. Bot. 56, 73–80. 10.1093/jxb/eri020 15533878

[B116] Marri L. Trost P. Trivelli X. Gonnelli L. Pupillo P. Sparla F. (2008). Spontaneous assembly of photosynthetic supramolecular complexes as mediated by the intrinsically unstructured protein CP12. J. Biol. Chem. 283, 1831–1838. 10.1074/jbc.M705650200 17947231

[B117] Marri L. Zaffagnini M. Collin V. Issakidis-Bourguet E. Lemaire S. D. Pupillo P.. (2009). Prompt and easy activation by specific thioredoxins of Calvin cycle enzymes of *Arabidopsis thaliana* associated in the GAPDH/CP12/PRK supramolecular complex. Mol. Plant 2, 259–269. 10.1093/mp/ssn061 19825612

[B118] Marsh J. A. Teichmann S. A. (2010). How do proteins gain new domains? Genome Biol. 11, 126. 10.1186/gb-2010-11-7-126 20630117 PMC2926777

[B119] Martino-Catt S. Ort D. R. (1992). Low temperature interrupts circadian regulation of transcriptional activity in chilling-sensitive plants. Proc. Natl. Acad. Sci. U.S.A. 89, 3731–3735. 10.1073/pnas.89.9.3731 1570291 PMC525564

[B120] Matsumura H. Kai A. Maeda T. Tamoi M. Satoh A. Tamura H.. (2011). Structure basis for the regulation of glyceraldehyde-3-phosphate dehydrogenase activity via the intrinsically disordered protein CP12. Structure 19, 1846–1854. 10.1016/j.str.2011.08.016 22153507

[B121] Meszaros B. Tompa P. Simon I. Dosztanyi Z. (2007). Molecular principles of the interactions of disordered proteins. J. Mol. Biol. 372, 549–561. 10.1016/j.jmb.2007.07.004 17681540

[B122] Mileo E. Lorenzi M. Erales J. Lignon S. Puppo C. Le Breton N.. (2013). Dynamics of the intrinsically disordered protein CP12 in its association with GAPDH in the green alga *Chlamydomonas reinhardtii*: a fuzzy complex. Mol. Biosyst. 9, 2869–2876. 10.1039/c3mb70190e 24056937

[B123] Mittag T. Kay L. E. Forman-Kay J. D. (2010). Protein dynamics and conformational disorder in molecular recognition. J. Mol. Recognit. 23, 105–116. 10.1002/jmr.961 19585546

[B124] Mizianty M. J. Uversky V. Kurgan L. (2014). Prediction of intrinsic disorder in proteins using MFDp2. Methods Mol. Biol. 1137, 147–162. 10.1007/978-1-4939-0366-5_11 24573480

[B125] Mohan A. Oldfield C. J. Radivojac P. Vacic V. Cortese M. S. Dunker A. K.. (2006). Analysis of molecular recognition features (MoRFs). J. Mol. Biol. 362, 1043–1059. 10.1016/j.jmb.2006.07.087 16935303

[B126] Moparthi S. B. Thieulin-Pardo G. De Torres J. Ghenuche P. Gontero B. Wenger J. (2015). FRET analysis of CP12 structural interplay by GAPDH and PRK. Biochem. Biophys. Res. Commun. 10.1016/j.bbrc.2015.01.135 25666947

[B127] Moparthi S. B. Thieulin-Pardo G. Mansuelle P. Rigneault H. Gontero B. Wenger J. (2014). Conformational modulation and hydrodynamic radii of CP12 protein and its complexes probed by fluorescence correlation spectroscopy. FEBS J. 281, 3206–3217. 10.1111/febs.12854 24863370

[B128] Moreau V. H. Da Silva A. C. Siloto R. M. Valente A. P. Leite A. Almeida F. C. (2004). The bZIP region of the plant transcription factor opaque-2 forms stable homodimers in solution and retains its helical structure upon subunit dissociation. Biochemistry 43, 4862–4868. 10.1021/bi035905e 15096055

[B129] Mueller-Cajar O. Stotz M. Bracher A. (2014). Maintaining photosynthetic CO_2_ fixation via protein remodelling: the Rubisco activases. Photosyn. Res. 119, 191–201. 10.1007/s11120-013-9819-0 23543331

[B130] Mueller-Cajar O. Stotz M. Wendler P. Hartl F. U. Bracher A. Hayer-Hartl M. (2011). Structure and function of the AAA^+^ protein CbbX, a red-type Rubisco activase. Nature 479, 194–199. 10.1038/nature10568 22048315

[B131] Munoz-Bertomeu J. Cascales-Minana B. Alaiz M. Segura J. Ros R. (2010). A critical role of plastidial glycolytic glyceraldehyde-3-phosphate dehydrogenase in the control of plant metabolism and development. Plant Signal. Behav. 5, 67–69. 10.4161/psb.5.1.10200 20592814 PMC2835963

[B132] Murase K. Hirano Y. Sun T. P. Hakoshima T. (2008). Gibberellin-induced DELLA recognition by the gibberellin receptor GID1. Nature 456, 459–463. 10.1038/nature07519 19037309

[B133] Neuwald A. F. Aravind L. Spouge J. L. Koonin E. V. (1999). AAA^+^: a class of chaperone-like ATPases associated with the assembly, operation, and disassembly of protein complexes. Genome Res. 9, 27–43. 9927482

[B134] Oldfield C. J. Cheng Y. Cortese M. S. Romero P. Uversky V. N. Dunker A. K. (2005). Coupled folding and binding with alpha-helix-forming molecular recognition elements. Biochemistry 44, 12454–12470. 10.1021/bi050736e 16156658

[B135] Olsen A. N. Ernst H. A. Leggio L. L. Skriver K. (2005). NAC transcription factors: structurally distinct, functionally diverse. Trends Plant Sci. 10, 79–87. 10.1016/j.tplants.2004.12.010 15708345

[B136] Ooka H. Satoh K. Doi K. Nagata T. Otomo Y. Murakami K.. (2003). Comprehensive analysis of NAC family genes in *Oryza sativa* and *Arabidopsis thaliana*. DNA Res. 10, 239–247. 10.1093/dnares/10.6.239 15029955

[B137] Ozturk N. Song S. H. Ozgur S. Selby C. P. Morrison L. Partch C.. (2007). Structure and function of animal cryptochromes. Cold Spring Harb. Symp. Quant. Biol. 72, 119–131. 10.1101/sqb.2007.72.015 18419269

[B138] Pancsa R. Tompa P. (2012). Structural disorder in eukaryotes. PLoS ONE 7:e34687. 10.1371/journal.pone.0034687 22496841 PMC3320622

[B139] Panda A. Ghosh T. C. (2014). Prevalent structural disorder carries signature of prokaryotic adaptation to oxic atmosphere. Gene 548, 134–141. 10.1016/j.gene.2014.07.002 24999584

[B140] Partch C. L. Clarkson M. W. Ozgur S. Lee A. L. Sancar A. (2005). Role of structural plasticity in signal transduction by the cryptochrome blue-light photoreceptor. Biochemistry 44, 3795–3805. 10.1021/bi047545g 15751956

[B141] Patil A. Nakamura H. (2006). Disordered domains and high surface charge confer hubs with the ability to interact with multiple proteins in interaction networks. FEBS Lett. 580, 2041–2045. 10.1016/j.febslet.2006.03.003 16542654

[B142] Pearce F. G. (2006). Catalytic by-product formation and ligand binding by ribulose bisphosphate carboxylases from different phylogenies. Biochem. J. 399, 525–534. 10.1042/BJ20060430 16822231 PMC1615894

[B143] Perchorowicz J. T. Raynes D. A. Jensen R. G. (1981). Light limitation of photosynthesis and activation of ribulose bisphosphate carboxylase in wheat seedlings. Proc. Natl. Acad. Sci. U.S.A. 78, 2985–2989. 10.1073/pnas.78.5.2985 16593018 PMC319484

[B144] Peschel N. Chen K. F. Szabo G. Stanewsky R. (2009). Light-dependent interactions between the *Drosophila* circadian clock factors cryptochrome, jetlag, and timeless. Curr. Biol. 19, 241–247. 10.1016/j.cub.2008.12.042 19185492

[B145] Petersen J. Brinkmann H. Cerff R. (2003). Origin, evolution, and metabolic role of a novel glycolytic GAPDH enzyme recruited by land plant plastids. J. Mol. Evol. 57, 16–26. 10.1007/s00239-002-2441-y 12962302

[B146] Petersen J. Teich R. Becker B. Cerff R. Brinkmann H. (2006). The GapA/B gene duplication marks the origin of Streptophyta (charophytes and land plants). Mol. Biol. Evol. 23, 1109–1118. 10.1093/molbev/msj123 16527864

[B147] Pietrosemoli N. Garcia-Martin J. A. Solano R. Pazos F. (2013). Genome-wide analysis of protein disorder in *Arabidopsis thaliana*: implications for plant environmental adaptation. PLoS ONE 8:e55524. 10.1371/journal.pone.0055524 23408995 PMC3567104

[B148] Podust L. M. Krezel A. M. Kim Y. (2001). Crystal structure of the CCAAT box/enhancer-binding protein beta activating transcription factor-4 basic leucine zipper heterodimer in the absence of DNA. J. Biol. Chem. 276, 505–513. 10.1074/jbc.M005594200 11018027

[B149] Pohlmeyer K. Paap B. K. Soll J. Wedel N. (1996). CP12: a small nuclear-encoded chloroplast protein provides novel insights into higher-plant GAPDH evolution. Plant Mol. Biol. 32, 969–978. 10.1007/BF00020493 8980547

[B150] Portis A. R. Jr. (2003). Rubisco activase - Rubisco's catalytic chaperone. Photosyn. Res. 75, 11–27. 10.1023/A:1022458108678 16245090

[B151] Portis A. R. Jr. Li C. Wang D. Salvucci M. E. (2008). Regulation of Rubisco activase and its interaction with Rubisco. J. Exp. Bot. 59, 1597–1604. 10.1093/jxb/erm240 18048372

[B152] Portis A. R. Salvucci M. E. Ogren W. L. (1986). Activation of ribulosebisphosphate carboxylase/oxygenase at physiological CO_2_ and ribulosebisphosphate concentrations by Rubisco activase. Plant Physiol. 82, 967–971. 10.1104/pp.82.4.967 16665175 PMC1056242

[B153] Pupillo P. Giuliani Piccari G. (1975). The reversible depolymerization of spinach chloroplast glyceraldehyde-phosphate dehydrogenase. Interaction with nucleotides and dithiothreitol. Eur. J. Biochem. 51, 475–482. 10.1111/j.1432-1033.1975.tb03947.x 238837

[B154] Pupillo P. Piccari G. G. (1973). The effect of NADP on the subunit structure and activity of spinach chloroplast glyceraldehyde-3-phosphate dehydrogenase. Arch. Biochem. Biophys. 154, 324–331. 10.1016/0003-9861(73)90064-7 4144055

[B155] Qian J. Rodermel S. R. (1993). Ribulose-1,5-bisphosphate carboxylase/oxygenase activase cDNAs from *Nicotiana tabacum*. Plant Physiol. 102, 683–684. 10.1104/pp.102.2.683 8108517 PMC158830

[B156] Raven J. A. (2013). Rubisco: still the most abundant protein of Earth? New Phytol. 198, 1–3. 10.1111/nph.12197 23432200

[B157] Robbens S. Petersen J. Brinkmann H. Rouze P. Van De Peer Y. (2007). Unique regulation of the Calvin cycle in the ultrasmall green alga *Ostreococcus*. J. Mol. Evol. 64, 601–604. 10.1007/s00239-006-0159-y 17457634

[B158] Romero P. Obradovic Z. Kissinger C. R. Villafranca J. E. Garner E. Guilliot S. (1998). Thousands of proteins likely to have long disordered regions. Pac. Symp. Biocomput. 3, 437–448. Available online at: http://citeseerx.ist.psu.edu/viewdoc/summary?doi=10.1.1.132.4594 9697202

[B159] Rundle S. J. Zielinski R. E. (1991). Organization and expression of two tandemly oriented genes encoding ribulosebisphosphate carboxylase/oxygenase activase in barley. J. Biol. Chem. 266, 4677–4685. 2002016

[B160] Rushton P. J. Bokowiec M. T. Han S. Zhang H. Brannock J. F. Chen X.. (2008). Tobacco transcription factors: novel insights into transcriptional regulation in the *Solanaceae*. Plant Physiol. 147, 280–295. 10.1104/pp.107.114041 18337489 PMC2330323

[B161] Saha T. Kar R. K. Sa G. (2014). Structural and sequential context of p53: a review of experimental and theoretical evidence. Prog. Biophys. Mol. Biol. 117, 250–263. 10.1016/j.pbiomolbio.2014.12.002 25550083

[B162] Salminen A. Ojala J. Kaarniranta K. Hiltunen M. Soininen H. (2011). Hsp90 regulates tau pathology through co-chaperone complexes in Alzheimer's disease. Prog. Neurobiol. 93, 99–110. 10.1016/j.pneurobio.2010.10.006 21056617

[B163] Salvucci M. E. (2004). Potential for interactions between the carboxy- and amino-termini of Rubisco activase subunits. FEBS Lett. 560, 205–209. 10.1016/S0014-5793(04)00111-5 14988023

[B164] Salvucci M. E. Rajagopalan K. Sievert G. Haley B. E. Watt D. S. (1993). Photoaffinity labeling of ribulose-1,5-bisphosphate carboxylase/oxygenase activase with ATP gamma-benzophenone. Identification of the ATP gamma-phosphate binding domain. J. Biol. Chem. 268, 14239–14244. 8314787

[B165] Salvucci M. E. Van De Loo F. J. Stecher D. (2003). Two isoforms of Rubisco activase in cotton, the products of separate genes not alternative splicing. Planta 216, 736–744. 10.1007/s00425-002-0923-1 12624760

[B166] Sancar A. (2004). Photolyase and cryptochrome blue-light photoreceptors. Adv. Protein Chem. 69, 73–100. 10.1016/S0065-3233(04)69003-6 15588840

[B167] Sanchez C. Vielba J. M. Ferro E. Covelo G. Sole A. Abarca D.. (2007). Two SCARECROW-LIKE genes are induced in response to exogenous auxin in rooting-competent cuttings of distantly related forest species. Tree Physiol. 27, 1459–1470. 10.1093/treephys/27.10.1459 17669736

[B168] Scagliarini S. Trost P. Pupillo P. (1998). The non-regulatory isoform of NADP(H)-glyceraldehyde 3-phosphate dehydrogenase from spinach chloroplasts. J. Exp. Bot. 49, 1307–1315. 10.1093/jxb/49.325.1307

[B169] Scheibe R. Wedel N. Vetter S. Emmerlich V. Sauermann S. M. (2002). Co-existence of two regulatory NADP-glyceraldehyde 3-P dehydrogenase complexes in higher plant chloroplasts. Eur. J. Biochem. 269, 5617–5624. 10.1046/j.1432-1033.2002.03269.x 12423361

[B170] Sedzik J. Kirschner D. A. (1992). Is myelin basic protein crystallizable? Neurochem. Res. 17, 157–166. 10.1007/BF00966794 1371603

[B171] Seo P. J. Kim M. J. Song J. S. Kim Y. S. Kim H. J. Park C. M. (2010). Proteolytic processing of an *Arabidopsis* membrane-bound NAC transcription factor is triggered by cold-induced changes in membrane fluidity. Biochem. J. 427, 359–367. 10.1042/BJ20091762 20156199

[B172] Seo P. J. Park C. M. (2010). A membrane-bound NAC transcription factor as an integrator of biotic and abiotic stress signals. Plant Signal. Behav. 5, 481–483. 10.4161/psb.11083 20139739 PMC7080469

[B173] Shen J. B. Ogren W. L. (1992). Alteration of spinach ribulose-1,5-bisphosphate carboxylase/oxygenase activase activities by site-directed mutagenesis. Plant Physiol. 99, 1201–1207. 10.1104/pp.99.3.1201 16668989 PMC1080603

[B174] Singh P. Kaloudas D. Raines C. A. (2008). Expression analysis of the *Arabidopsis* CP12 gene family suggests novel roles for these proteins in roots and floral tissues. J. Exp. Bot. 59, 3975–3985. 10.1093/jxb/ern236 18974062 PMC2576635

[B175] Sirover M. A. (1999). New insights into an old protein: the functional diversity of mammalian glyceraldehyde-3-phosphate dehydrogenase. Biochim. Biophys. Acta 1432, 159–184. 10.1016/S0167-4838(99)00119-3 10407139

[B176] Sirover M. A. (2011). On the functional diversity of glyceraldehyde-3-phosphate dehydrogenase: biochemical mechanisms and regulatory control. Biochim. Biophys. Acta 1810, 741–751. 10.1016/j.bbagen.2011.05.010 21640161

[B177] Sparla F. Fermani S. Falini G. Zaffagnini M. Ripamonti A. Sabatino P.. (2004). Coenzyme site-directed mutants of photosynthetic A_4_-GAPDH show selectively reduced NADPH-dependent catalysis, similar to regulatory A_2_B_2_-GAPDH inhibited by oxidized thioredoxin. J. Mol. Biol. 340, 1025–1037. 10.1016/j.jmb.2004.06.005 15236965

[B178] Sparla F. Pupillo P. Trost P. (2002). The C-terminal extension of glyceraldehyde-3-phosphate dehydrogenase subunit B acts as an autoinhibitory domain regulated by thioredoxins and nicotinamide adenine dinucleotide. J. Biol. Chem. 277, 44946–44952. 10.1074/jbc.M206873200 12270927

[B179] Sparla F. Zaffagnini M. Wedel N. Scheibe R. Pupillo P. Trost P. (2005). Regulation of photosynthetic GAPDH dissected by mutants. Plant Physiol. 138, 2210–2219. 10.1104/pp.105.062117 16055685 PMC1183408

[B180] Srivastava R. Deng Y. Howell S. H. (2014). Stress sensing in plants by an ER stress sensor/transducer, bZIP28. Front. Plant Sci. 5:59. 10.3389/fpls.2014.00059 24616727 PMC3935173

[B181] Srivastava R. Deng Y. Shah S. Rao A. G. Howell S. H. (2013). BINDING PROTEIN is a master regulator of the endoplasmic reticulum stress sensor/transducer bZIP28 in Arabidopsis. Plant Cell 25, 1416–1429. 10.1105/tpc.113.110684 23624714 PMC3663277

[B182] Stanley D. N. Raines C. A. Kerfeld C. A. (2013). Comparative analysis of 126 cyanobacterial genomes reveals evidence of functional diversity among homologs of the redox-regulated CP12 protein. Plant Physiol. 161, 824–835. 10.1104/pp.112.210542 23184231 PMC3561022

[B183] Steiner E. Efroni I. Gopalraj M. Saathoff K. Tseng T. S. Kieffer M.. (2012). The *Arabidopsis* O-linked N-acetylglucosamine transferase SPINDLY interacts with class I TCPs to facilitate cytokinin responses in leaves and flowers. Plant Cell 24, 96–108. 10.1105/tpc.111.093518 22267487 PMC3289577

[B184] Stotz M. Mueller-Cajar O. Ciniawsky S. Wendler P. Hartl F. U. Bracher A.. (2011). Structure of green-type Rubisco activase from tobacco. Nat. Struct. Mol. Biol. 18, 1366–1370. 10.1038/nsmb.2171 22056769

[B185] Sun X. Jones W. T. Harvey D. Edwards P. J. Pascal S. M. Kirk C.. (2010). N-terminal domains of DELLA proteins are intrinsically unstructured in the absence of interaction with GID1/gibberellic acid receptors. J. Biol. Chem. 285, 11557–11571. 10.1074/jbc.M109.027011 20103592 PMC2857034

[B186] Sun X. Jones W. T. Rikkerink E. H. (2012). GRAS proteins: the versatile roles of intrinsically disordered proteins in plant signalling. Biochem. J. 442, 1–12. 10.1042/BJ20111766 22280012

[B187] Sun X. Rikkerink E. H. Jones W. T. Uversky V. N. (2013). Multifarious roles of intrinsic disorder in proteins illustrate its broad impact on plant biology. Plant Cell 25, 38–55. 10.1105/tpc.112.106062 23362206 PMC3584547

[B188] Sun X. Xue B. Jones W. T. Rikkerink E. Dunker A. K. Uversky V. N. (2011). A functionally required unfoldome from the plant kingdom: intrinsically disordered N-terminal domains of GRAS proteins are involved in molecular recognition during plant development. Plant Mol. Biol. 77, 205–223. 10.1007/s11103-011-9803-z 21732203

[B189] Taoka K. Yanagimoto Y. Daimon Y. Hibara K. Aida M. Tasaka M. (2004). The NAC domain mediates functional specificity of CUP-SHAPED COTYLEDON proteins. Plant J. 40, 462–473. 10.1111/j.1365-313X.2004.02238.x 15500463

[B190] Tian C. Wan P. Sun S. Li J. Chen M. (2004). Genome-wide analysis of the GRAS gene family in rice and *Arabidopsis*. Plant Mol. Biol. 54, 519–532. 10.1023/B:PLAN.0000038256.89809.57 15316287

[B191] To K. Y. Suen D. F. Chen S. C. (1999). Molecular characterization of ribulose-1,5-bisphosphate carboxylase/oxygenase activase in rice leaves. Planta 209, 66–76. 10.1007/s004250050607 10467032

[B192] Tompa P. (2002). Intrinsically unstructured proteins. Trends Biochem. Sci. 27, 527–533. 10.1016/S0968-0004(02)02169-2 12368089

[B193] Tompa P. (2009). Structural disorder in amyloid fibrils: its implication in dynamic interactions of proteins. FEBS J. 276, 5406–5415. 10.1111/j.1742-4658.2009.07250.x 19712107

[B194] Tompa P. Fuxreiter M. (2008). Fuzzy complexes: polymorphism and structural disorder in protein-protein interactions. Trends Biochem. Sci. 33, 2–8. 10.1016/j.tibs.2007.10.003 18054235

[B195] Triezenberg S. J. (1995). Structure and function of transcriptional activation domains. Curr. Opin. Genet. Dev. 5, 190–196. 10.1016/0959-437X(95)80007-7 7613088

[B196] Trost P. Fermani S. Marri L. Zaffagnini M. Falini G. Scagliarini S.. (2006). Thioredoxin-dependent regulation of photosynthetic glyceraldehyde-3-phosphate dehydrogenase: autonomous vs. CP12-dependent mechanisms. Photosyn. Res. 89, 1–13. 10.1007/s11120-006-9099-z 17031544

[B197] Trost P. Pupillo P. (1993). Inhibition of spinach D-glyceraldehyde 3-phosphate: NADP+ oxidoreductase (nonphosphorylating) by adenylate compounds: the effect of dead-end inhibitors on a steady state random reaction mechanism. Arch. Biochem. Biophys. 306, 76–82. 10.1006/abbi.1993.1483 8215424

[B198] Uversky V. N. (2002). What does it mean to be natively unfolded? Eur. J. Biochem. 269, 2–12. 10.1046/j.0014-2956.2001.02649.x 11784292

[B199] Uversky V. N. (2009). Intrinsic disorder in proteins associated with neurodegenerative diseases. Front. Biosci. 14, 5188–5238. 10.2741/3594 19482612

[B200] Uversky V. N. (2010). The mysterious unfoldome: structureless, underappreciated, yet vital part of any given proteome. J. Biomed. Biotechnol. 2010:568068. 10.1155/2010/568068 20011072 PMC2789583

[B201] Uversky V. N. (2011). Multitude of binding modes attainable by intrinsically disordered proteins: a portrait gallery of disorder-based complexes. Chem. Soc. Rev. 40, 1623–1634. 10.1039/C0CS00057D 21049125

[B202] Uversky V. N. (2013). The most important thing is the tail: multitudinous functionalities of intrinsically disordered protein termini. FEBS Lett. 587, 1891–1901. 10.1016/j.febslet.2013.04.042 23665034

[B203] Uversky V. N. Dunker A. K. (2010). Understanding protein non-folding. Biochim. Biophys. Acta 1804, 1231–1264. 10.1016/j.bbapap.2010.01.017 20117254 PMC2882790

[B204] Uversky V. N. Gillespie J. R. Fink A. L. (2000). Why are “natively unfolded” proteins unstructured under physiologic conditions? Proteins 41, 415–427. 10.1002/1097-0134(20001115)41:3<415::AID-PROT130>3.0.CO;2-7 11025552

[B205] Vacic V. Oldfield C. J. Mohan A. Radivojac P. Cortese M. S. Uversky V. N.. (2007). Characterization of molecular recognition features, MoRFs, and their binding partners. J. Proteome Res. 6, 2351–2366. 10.1021/pr0701411 17488107 PMC2570643

[B206] Valsecchi I. Guittard-Crilat E. Maldiney R. Habricot Y. Lignon S. Lebrun R.. (2013). The intrinsically disordered C-terminal region of *Arabidopsis thaliana* TCP8 transcription factor acts both as a transactivation and self-assembly domain. Mol. Biosyst. 9, 2282–2295. 10.1039/c3mb70128j 23760157

[B207] Van De Loo F. J. Salvucci M. E. (1996). Activation of ribulose-1,5-biphosphate carboxylase/oxygenase (Rubisco) involves Rubisco activase Trp16. Biochemistry 35, 8143–8148. 10.1021/bi9604901 8679566

[B208] Vinson C. R. Hai T. Boyd S. M. (1993). Dimerization specificity of the leucine zipper-containing bZIP motif on DNA binding: prediction and rational design. Genes Dev. 7, 1047–1058. 10.1101/gad.7.6.1047 8504929

[B209] Viola I. L. Reinheimer R. Ripoll R. Manassero N. G. Gonzalez D. H. (2012). Determinants of the DNA binding specificity of class I and class II TCP transcription factors. J. Biol. Chem. 287, 347–356. 10.1074/jbc.M111.256271 22074922 PMC3249086

[B210] Viola I. L. Uberti Manassero N. G. Ripoll R. Gonzalez D. H. (2011). The *Arabidopsis* class I TCP transcription factor AtTCP11 is a developmental regulator with distinct DNA-binding properties due to the presence of a threonine residue at position 15 of the TCP domain. Biochem. J. 435, 143–155. 10.1042/BJ20101019 21241251

[B211] Wang D. Portis A. R. Jr. (2006). Increased sensitivity of oxidized large isoform of ribulose-1,5-bisphosphate carboxylase/oxygenase (rubisco) activase to ADP inhibition is due to an interaction between its carboxyl extension and nucleotide-binding pocket. J. Biol. Chem. 281, 25241–25249. 10.1074/jbc.M604756200 16822862

[B212] Wang H. Ma L. G. Li J. M. Zhao H. Y. Deng X. W. (2001). Direct interaction of *Arabidopsis* cryptochromes with COP1 in light control development. Science 294, 154–158. 10.1126/science.1063630 11509693

[B213] Watillon B. Kettmann R. Boxus P. Burny A. (1993). Developmental and circadian pattern of rubisco activase mRNA accumulation in apple plants. Plant Mol. Biol. 23, 501–509. 10.1007/BF00019298 8219085

[B214] Werneke J. M. Chatfield J. M. Ogren W. L. (1988). Catalysis of ribulosebisphosphate carboxylase/oxygenase activation by the product of a Rubisco activase cDNA clone expressed in *Escherichia coli*. Plant Physiol. 87, 917–920. 10.1104/pp.87.4.917 16666245 PMC1054869

[B215] Werneke J. M. Chatfield J. M. Ogren W. L. (1989). Alternative mRNA splicing generates the two ribulosebisphosphate carboxylase/oxygenase activase polypeptides in spinach and *Arabidopsis*. Plant Cell 1, 815–825. 10.1105/tpc.1.8.815 2535524 PMC159819

[B216] Wolosiuk R. A. Buchanan B. B. (1976). Studies on the regulation of chloroplast NADP-linked glyceraldehyde-3-phosphate dehydrogenase. J. Biol. Chem. 251, 6456–6461. 10297

[B217] Wolosiuk R. A. Buchanan B. B. (1978). Regulation of chloroplast phosphoribulokinase by the ferredoxin/thioredoxin system. Arch. Biochem. Biophys. 189, 97–101. 10.1016/0003-9861(78)90119-4 213020

[B218] Wright P. E. Dyson H. J. (1999). Intrinsically unstructured proteins: re-assessing the protein structure-function paradigm. J. Mol. Biol. 293, 321–331. 10.1006/jmbi.1999.3110 10550212

[B219] Xue B. Ganti K. Rabionet A. Banks L. Uversky V. N. (2014). Disordered interactome of human papillomavirus. Curr. Pharm. Des. 20, 1274–1292. 10.2174/13816128113199990072 23713779

[B220] Yang H. Q. Tang R. H. Cashmore A. R. (2001). The signaling mechanism of *Arabidopsis* CRY1 involves direct interaction with COP1. Plant Cell 13, 2573–2587. 10.1105/tpc.13.12.2573 11752373 PMC139474

[B221] Yang H. Q. Wu Y. J. Tang R. H. Liu D. Liu Y. Cashmore A. R. (2000). The C-termini of *Arabidopsis* cryptochromes mediate a constitutive light response. Cell 103, 815–827. 10.1016/S0092-8674(00)00184-7 11114337

[B222] Yin Z. Meng F. Song H. Wang X. Xu X. Yu D. (2010). Expression quantitative trait loci analysis of two genes encoding rubisco activase in soybean. Plant Physiol. 152, 1625–1637. 10.1104/pp.109.148312 20032079 PMC2832260

[B223] Yoon M. K. Shin J. Choi G. Choi B. S. (2006). Intrinsically unstructured N-terminal domain of bZIP transcription factor HY5. Proteins 65, 856–866. 10.1002/prot.21089 17001643

[B224] Yruela I. Contreras-Moreira B. (2012). Protein disorder in plants: a view from the chloroplast. BMC Plant Biol. 12:165. 10.1186/1471-2229-12-165 22970728 PMC3460767

[B225] Yruela I. Contreras-Moreira B. (2013). Genetic recombination is associated with intrinsic disorder in plant proteomes. BMC Genomics 14:772. 10.1186/1471-2164-14-772 24206529 PMC3828576

[B226] Yu X. Liu H. Klejnot J. Lin C. (2010). The cryptochrome blue light receptors. Arabidopsis Book 8:e0135. 10.1199/tab.0135 21841916 PMC3155252

[B227] Yu X. Shalitin D. Liu X. Maymon M. Klejnot J. Yang H.. (2007). Derepression of the NC80 motif is critical for the photoactivation of *Arabidopsis* CRY2. Proc. Natl. Acad. Sci. U.S.A. 104, 7289–7294. 10.1073/pnas.0701912104 17438275 PMC1855427

[B228] Zarzycki J. Axen S. D. Kinney J. N. Kerfeld C. A. (2013). Cyanobacterial-based approaches to improving photosynthesis in plants. J. Exp. Bot. 64, 787–798. 10.1093/jxb/ers294 23095996

[B229] Zhang N. Kallis R. P. Ewy R. G. Portis A. R. Jr. (2002). Light modulation of Rubisco in *Arabidopsis* requires a capacity for redox regulation of the larger Rubisco activase isoform. Proc. Natl. Acad. Sci. U.S.A. 99, 3330–3334. 10.1073/pnas.042529999 11854454 PMC122518

[B230] Zhang N. Portis A. R. Jr. (1999). Mechanism of light regulation of Rubisco: a specific role for the larger Rubisco activase isoform involving reductive activation by thioredoxin-*f*. Proc. Natl. Acad. Sci. U.S.A. 96, 9438–9443. 10.1073/pnas.96.16.9438 10430961 PMC17801

[B231] Zhang N. Schürmann P. Portis A. Jr. (2001). Characterization of the regulatory function of the 46-kDa isoform of Rubisco activase from *Arabidopsis*. Photosyn. Res. 68, 29–37. 10.1023/A:1011845506196 16228326

[B232] Zoltowski B. D. Vaidya A. T. Top D. Widom J. Young M. W. Crane B. R. (2011). Structure of full-length *Drosophila* cryptochrome. Nature 480, 396–399. 10.1038/nature10618 22080955 PMC3240699

[B233] Zuo Z. Liu H. Liu B. Liu X. Lin C. (2011). Blue light-dependent interaction of CRY2 with SPA1 regulates COP1 activity and floral initiation in *Arabidopsis*. Curr. Biol. 21, 841–847. 10.1016/j.cub.2011.03.048 21514160 PMC3150455

